# Germline deletion of *Rgs2* and/or *Rgs5* in male mice does not exacerbate left ventricular remodeling induced by subchronic isoproterenol infusion

**DOI:** 10.14814/phy2.70178

**Published:** 2025-01-02

**Authors:** Shelby Dahlen, Ipsita Mohanty, Bo Sun, Sanjana Nallapaneni, Patrick Osei‐Owusu

**Affiliations:** ^1^ Department of Physiology & Biophysics Case Western Reserve University School of Medicine Cleveland Ohio USA; ^2^ Department of Pharmacology & Physiology Drexel University College of Medicine Philadelphia Pennsylvania USA

**Keywords:** adrenergic receptors, cardiac physiology, cardiomyopathy, G protein signaling, RGS

## Abstract

Sympathoexcitation is a hallmark of heart failure, with sustained β‐adrenergic receptor (βAR)‐G protein signaling activation. βAR signaling is modulated by regulator of G protein signaling (RGS) proteins. Previously, we reported that Gα_i/o_ regulation by RGS2 or RGS5 is key to ventricular rhythm regulation, while the dual loss of both RGS proteins results in left ventricular (LV) dilatation and dysfunction. Here, we tested whether sustained βAR stimulation with isoproterenol (ISO, 30 mg/kg/day, 3 days) exacerbates LV remodeling in male mice with germline deletion of *Rgs2* and/or *Rgs5*. *Rgs2 KO and Rgs2/5* dbKO mice showed LV dilatation at baseline, which was unchanged by ISO. *Rgs2* or *Rgs5 deletion decreased Rgs1* expression, whereas *Rgs5* deletion increased *Rgs4* expression. ISO induced cardiac hypertrophy and interstitial fibrosis in *Rgs2/5* dbKO mice without increasing cardiomyocyte size or LV dilation but increased expression of cardiac fetal gene *Nppa*, α‐actinin, and Ca^2+^‐/calmodulin‐dependent kinase II. Single *Rgs2* and *Rgs5* KO mice had markedly increased CD45^+^ cells, whereas tissue from *Rgs5* KO mice showed increased CD68^+^ cells, as revealed by immunohistochemistry. The results together indicate that ventricular remodeling due to *Rgs2* and/or *Rgs5* deletion is associated with augmented myocardial immune cell presence but is not exacerbated by sustained βAR stimulation.

## Graphical abstract

Regulation of Gα_i/o_ by RGS2 and RGS5 of the R4 family of RGS proteins is key to homeostatic control of cardiac structure and function by adenylyl cyclase (AC)‐mediated cAMP signaling downstream of β‐adrenergic receptor (βAR), such that the absence of RGS2, RGS5, or both RGS proteins (*Rgs2* KO, *Rgs5* KO, and *Rgs2/5* dbKO) leads to compensatory changes in the expression of other R4 RGS proteins (RGS1‐5) and biomarkers of maladaptive cardiac remodeling and fibrosis (*Nppa*, *Col3a1*, CaMKII, α‐actinin), increased interstitial fibrosis, left ventricular (LV) dilatation, and decreased contractile function (%EF and %FS). And, sustained βAR stimulation with systemic isoproterenol (ISO) infusion in *Rgs2* KO or *Rgs5* KO mice increases cardiac immune cell infiltration (CD45^+^ & CD68^+^ cells, respectively) without exacerbating impaired cardiac function and LV dilatation.

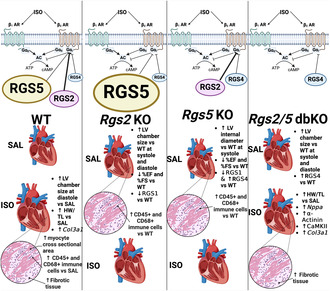

## 1 INTRODUCTION

Heart failure is a leading cause of morbidity and mortality worldwide and affects over 6 million adults in the United States (Tsao et al., 2023). Abnormally high sympathetic activity is a primary maladaptive autonomic nervous system activity in heart failure with reduced ejection fraction, as a compensatory response to a decline in cardiac output. Numerous studies have demonstrated that the cardiac β‐adrenergic receptor system is altered in chronic heart failure (Bristow et al., 1982; Engelhardt et al., 1996; Kiuchi et al., 1993; Lohse et al., 2003). As such, drugs that antagonize cardiac adrenergic (specifically, β‐adrenergic) receptors (aka β‐blockers) are a standard treatment for heart failure (Cadrin‐Tourigny et al., 2017; He et al., 2022; Investigators B‐BEoST., 2001; Packer et al., 1996). In failing hearts, the expression of the predominant β‐adrenergic receptor (βAR) in the myocardium, the G_s_‐coupled β_1_AR, is reduced by up to 50% (Engelhardt et al., 1996). Conversely, the expression of the G_s_‐ and G_i/o_‐coupled β_2_AR is unchanged, despite increased expression of Gα_i/o_ in the myocardium (Böhm et al., 1990). These changes in receptor expression disturb the balance between G_s_ and G_i/o_ influence on adenylyl cyclase activity and cAMP generation in the myocardium. In normal physiologic state, G protein signaling is closely regulated to ensure optimal duration and magnitude of cellular response to receptor stimulation (Syrovatkina et al., 2016). The loss or alteration of such modulation of heterotrimeric G protein signaling is implicated in several forms of heart disease including heart failure, thus making G proteins and their coupled receptors (GPCRs) the primary target of major classes of heart failure drugs. Enhanced understanding of the mechanisms by which various G protein signaling cascades are regulated during homeostasis, and how such regulatory mechanisms may be altered or lost as part of heart disease pathogenesis, is critical to identifying novel drug targets.

In their active conformation, GPCRs, including βAR in the myocardium, activate G protein signaling by acting as guanine nucleotide exchange factor to facilitate the exchange of GDP for GTP bound to the Gα subunit, thereby leading to subsequent separation of the GTP‐bound Gα from the Gβγ obligate dimer (Rockman & Lefkowitz, 2024; Wess, 1997). Conversely, G protein signaling is terminated upon hydrolysis of the bound GTP via the intrinsic GTPase activity of the Gα subunit, followed by rapid reassembly of the heterotrimer in a GDP‐bound state (Chidiac & Roy, 2003; Rockman & Lefkowitz, 2024). Signaling via heterotrimeric G proteins is finetuned by GTPase activating proteins (GAP), including members of the regulator of G protein signaling (RGS) protein family (Hepler & Gilman, 1992). GAP, including RGS proteins, facilitate the intrinsic GTPase activity of Gα subunits, thereby accelerating GTP hydrolysis and reassembly of the inactive, GDP‐bound G_αβγ_ for a new round of activation by an active GPCR (Chidiac & Roy, 2003). Roughly, 30 mammalian RGS2 proteins, classified into nine families, have been discovered to date (Bansal et al., 2007). Among these families, several members of the R4 family of RGS proteins, including RGS1‐5, are prominently expressed in the myocardium, with varying GAP selectivity towards G_q/11_ and G_i/o_ class G proteins (Hao et al., 2006; Riddle et al., 2005). Specifically, RGS2 and RGS5 have been reported to act as GAP toward G_i/o_‐coupled β_2_AR (Chakir et al., 2011; Hao et al., 2006; Zhang & Mende, 2014), and overexpression of RGS2 or RGS5 in human embryonic kidney (HEK 293) cells, and RGS2 in cultured adult mouse cardiomyocytes, suppresses β_2_AR‐G_i/o_‐mediated signaling activated with the nonspecific βAR agonist, isoproterenol (ISO) (Chakir et al., 2011). Conversely, deletion of either *Rgs2* or *Rgs5* increases susceptibility to cardiac hypertrophy and arrhythmia in mice (Jean‐Baptiste et al., 2005; Jones et al., 2012; Li et al., 2010; Nunn et al., 2010; Qin et al., 2012, 2016; Song et al., 2021; Takimoto et al., 2009; Tuomi et al., 2010). However, at basal state, cardiac structure and function, as assessed via echocardiography, have previously been reported to be inappreciably altered in both *Rgs2* and *Rgs5* KO mice relative to wild‐type (WT) cohorts (Ding et al., 2018; Heximer et al., 2003; Qin et al., 2012; Song et al., 2021; Takimoto et al., 2009).

Previously, we reported that mice dually lacking RGS2 and RGS5 (*Rgs2/5* dbKO) display sex‐related differences in cardiac structure and function, both at basal state and in response to surgery‐induced stress (Dahlen et al., 2022). Male *Rgs2/5* dbKO mice, unlike their female counterparts displayed left ventricular (LV) dilation at baseline and were susceptible to death from surgery‐induced stress (Dahlen et al., 2022). Additionally, LV cardiomyocytes (LVCM) from male *Rgs2/5* dbKO and *Rgs2* KO mice developed abnormal contractile and calcium transient rhythms upon pacing with electric field stimulation (EFS) or βAR‐mediated stimulation with ISO. These abnormalities were attributed, at least partly, to aberrant G_i/o_ activity and impaired calcium handling (Dahlen et al., 2022). In this study, we tested the hypothesis that LV remodeling resulting from the dual deletion of *Rgs2* and *Rgs5* exacerbates cardiac dysfunction due to acute elevation of circulating norepinephrine associated with sympathoexcitation, as a result of aberrant β_2_AR‐G_i/o_ signaling in the myocardium. Herein, we report that the loss of RGS2 and/or RGS5 leads to increased LV chamber size, accompanied by decreased contractile function and increased myocardial immune cell population at basal state; however, the abnormal structural and functional remodeling are unaffected by sustained βAR stimulation with ISO.

## 2 MATERIALS AND METHODS

### 2.1 Animals

Male mice (2–6 months old, ~28 g), including inbred wild type (WT) of the Charles River C57BL/6 genetic background, and global *Rgs2* KO, *Rgs5* KO, and *Rgs2/5* dbKO mice were used in this study. The generation of germline *Rgs2* KO, *Rgs5* KO, and *Rgs2/5* dbKO mice has been described previously (Cho et al., 2008; Dahlen et al., 2022; Oliveira‐dos‐Santos et al., 2000). Briefly, the traditional gene targeting method involving the use of mouse embryonic stem cell clones screened for bearing the targeted gene were injected into blastocysts that were in turn implanted in pseudo‐pregnant mice, followed by breeding of chimera mice until the achievement of germline transmission of the targeted gene. *Rgs2* KO mice were obtained from Dr. Kendall Blumer's group at Washington University School of Medicine in St. Louis, MO, with permission from Dr. Josef Penninger, whereas *Rgs5* KO mice were obtained from Dr. John Kehrl's group at the NIH (Cho et al., 2008; Oliveira‐dos‐Santos et al., 2000). Global *Rgs2/5* dbKO mice were derived from breeding *Rgs2 KO* and *Rgs5 KO* mice to generate double heterozygous KO (*Rgs2* ^*−/+*^,*Rgs5* ^*−/+*^) F1 offspring, which were in turn bred to obtain *Rgs2/5* dbKO mice, as previously described (Dahlen et al., 2022). All mice were provided access to food (standard mouse chow; 5P76/P3000, LabDiet, Richmond, IN) and water ad libitum in our institution's animal facility at 22 °C and a 12‐h light/dark cycle. The Institutional Animal Care and Use Committee at Case Western Reserve University approved the protocol for all animal experiments performed in this study, in accordance with the National Institutes of Health guidelines for the care and use of laboratory animals.

#### 2.1.1 Chemicals and reagents

For chronic infusion in mice, we used pharmaceutical‐grade isoproterenol (cat. # 1351005, Sigma Aldrich, St. Louis, MO) dissolved in sterile 0.9% saline (cat. #68099‐156, VWR, Radnor, PA). Isoflurane solution for inducing general anesthesia was purchased from Covetrus (cat. #029405, Covetrus, Dublin, Ohio). All other chemicals and reagents for the preparation of buffers and solutions were purchased from Sigma Aldrich (Sigma Aldrich, St. Louis, MO), except where indicated otherwise.

#### 2.1.2 Subchronic β‐adrenergic receptor stimulation

WT, *Rgs2* KO, *Rgs5* KO, and *Rgs2/5* dbKO male mice were randomly assigned to receive continuous intraperitoneal infusion of isoproterenol (ISO, 30 mg/kg/day) or vehicle (0.9% saline) at an identical infusion rate of 0.25 μL/h for 3 days using Alzet osmotic mini‐pump (cat. #2001, Braintree Scientific, Inc., Braintree, MA). Echocardiography data were acquired at the end of the 3‐day infusion period, followed by gravimetric analysis, immunohistochemistry, and histology on harvested tissue samples.

#### 2.1.3 Echocardiography

All echocardiography studies were performed using VisualSonics Vevo® 3100 imaging system (Scan head: MS400, 18–38 MHz, FUJIFILM VisualSonics, Inc., Bothell, WA). Anesthesia was induced for several minutes in an induction chamber (5% isoflurane mixed with air at a flow rate of 1.5 L/min). After placing the mouse in a supine position on a servo‐controlled heating pad equipped with ECG electrodes, anesthesia was maintained via inhalation of 1–1.5% isoflurane and air at a flow rate of 1.5 L/min using a nose mask. ECG signal was monitored throughout the procedure, and isoflurane level was lowered if the heart rate dropped below 450 bpm. After immobilizing the mouse on the heating pad with surgical tape, chest hair was removed with a depilation agent followed by the application of a layer of prewarmed ultrasound gel to the chest. Body temperature was monitored throughout the procedure by an inserted rectal probe and maintained at 37°C ± 1.5°C. Two‐dimensional (B‐mode) images in parasternal long‐axis view were used to position the probe at mid‐ventricular level for the acquisition of one‐dimensional M‐mode cine recordings. Standard measurements of interventricular septum (IVS), left ventricular internal diameter (LVID), and left ventricular posterior wall (LVPW) were performed during systole and diastole and calculated using the average of at least three beats. LV volume [μL] LVvol;d [7.0/(2.4 + LVID;d)] * LVID;d3, LVvol;s [7.0/(2.4 + LVID;s)] × LVID;s3, LV fractional shortening (FS) [%] [(LVID;d−LVID;s)/LVvol;d] × 100 and LV ejection fraction (EF) [%] [(LVvol;d−LVvol;s)/LVvol;d] × 100 were calculated from M‐mode measurements.

#### 2.1.4 Gravimetric analysis and tissue processing

Mice were anesthetized with 5% isoflurane and were euthanized via cervical dislocation. The heart was removed after thoracotomy and weighed. Lung and kidney weight, as well as tibia length, were measured. The heart was cut laterally into three pieces for further analyses. The apex and base of the heart were immediately frozen in dry ice and stored at −80°C. Midventricular slices were fixed in 10% formalin.

#### 2.1.5 Histological staining

Midventricular heart tissue samples were paraffin‐embedded, sectioned into 5‐μm slices, deparaffinized, and stained for Masson's trichrome using the services of the Tissue Resource Core at Case Western Reserve University School of Medicine. Brightfield photomicrographs were acquired under identical settings using Keyence All‐in‐One Fluorescence Microscope BZ‐X800 (Keyence Corp., Itasca, IL). The degree of interstitial fibrosis was scored by an investigator blinded to the genotype and treatment of the mice. An arbitrary scoring, ranging from 1 to 4, was used to determine the extensiveness of fibrosis. A score of 1 was assigned to a sample with the smallest area of trichrome staining, while a score of 4 was assigned to a sample with the largest area of staining in the field of view chosen at random. Average cell size was determined using 10 fields/heart of the trichrome photomicrographs taken at 40x magnification and analyzed with the freehand selection tool in NIH ImageJ software.

#### 2.1.6 In situ apoptosis detection by terminal deoxynucleotidyl transferase dUTP Nick end labeling (TUNEL)

Apoptosis was measured using CardioTACS In Situ Apoptosis Detection Kit (cat. #4827‐30‐K, R&D Systems, Inc., Minneapolis, MN) by following the instructions provided by the manufacturer. In brief, paraffinized tissue sections were heated at 57°C for 15 min, then deparaffinized and rehydrated using standard procedures, followed by Terminal deoxynucleotidyl Transferase (TdT) labeling and counterstaining with nuclear fast red. Labeled tissue slices were imaged at 40x using Keyence All‐in‐One Fluorescence Microscope BZ‐X800 (Keyence Corp., Itasca, IL). The number of TUNEL^+^ nuclei were counted and expressed as a percentage of total nuclei from 10 random fields per section.

#### 2.1.7 Immunohistochemistry

Immunohistochemical labeling of immune cells was performed using Vectastain® ABC‐HRP kit (cat. # PK‐4005 and PK‐4001, Vector Laboratories, Inc., Newark, CA) by following instructions provided by the manufacturer. Briefly, paraffinized midventricular tissue sections were heated at 57°C for 15 min, and then deparaffinized and rehydrated using standard procedures. For antigen retrieval, tissue sections on slides were placed in a boiling 8‐mM sodium citrate buffer (pH 6.0) for 10 min and allowed to cool to room temperature (RT), followed by several washes in distilled water and 0.05% Tween 20 in phosphate‐buffered saline (PBS‐T). The sections were then permeabilized in 0.1% Triton X‐100 in PBS for 10 min, washed with PBS‐T, and incubated in blocking buffer (1.5% stock blocking serum in PBS‐T) for 20 minutes. The sections were then covered in avidin solution for 15 min, rinsed with PBS‐T, then covered in biotin solution for 15 min (cat. # SP‐2001, Vector Laboratories, Inc., Newark, CA), followed by several rinses with PBS‐T and incubation with primary antibody solution against CD45 (cat. # AF114, 10 μg/mL, R&D Systems, Inc., Minneapolis, MN) or CD68 (cat. # MAB101141, 3 μg/mL, R&D Systems, Inc., Minneapolis, MN) for 1.5 h at RT in a humidity chamber. Following several rinses with PBS‐T, the sections were incubated in the appropriate secondary antibody solution (cat # PI‐9500‐1 or PI‐1000‐1, 1:200 dilution in 1.5% stock blocking serum in PBS‐T, Vector Laboratories, Inc., Newark, CA) for 45 min at RT in a humidity chamber. After streptavidin‐DAB staining, the slides were counterstained with nuclear fast red (cat # H‐3403‐500, Vector Laboratories, Inc., Newark, CA), dehydrated, cleared with xylene, and covered with mounting medium and coverglass. The slides were imaged at 40x using Keyence All‐in‐One Fluorescence Microscope BZ‐X800 (Keyence Corp., Itasca, IL). Immuno‐positive cells were counted from 10 random fields per section.

#### 2.1.8 Isolation and preparation of left ventricular cardiomyocytes from adult mouse hearts

Single cardiomyocytes were isolated using a Radnoti Langendorff apparatus (ADInstruments, Inc., Colorado Springs, CO). EDTA buffer containing (in mM) 130 NaCl, 5 KCl, 0.5 NaH_2_PO_4_, 10 HEPES, 10 glucose, 10 BDM (2,3‐butanedione monoxime), 10 taurine, and 5 EDTA (pH 7.8 with NaOH) was prepared and stored at 4°C up to 48 h. Perfusion buffer containing (in mM) 130 NaCl, 5 KCl, 0.5 NaH_2_PO_4_, 10 HEPES, 10 glucose, 10 BDM, 10 taurine, and 1 MgCl_2_ (pH 7.8 with NaOH) was prepared and stored at 4 °C, up to 48 h. Collagenase buffer containing 0.5 mg/mL collagenase type 2 (cat. # LS004174, Worthington Biochemical Corp., Lakewood, NJ), 0.5 mg/mL collagenase type 4 (Worthington Biochemical Corp., Lakewood, NJ), and 0.05 mg/mL protease XIV (cat. # P5147‐1G, Sigma Aldrich, St. Louis, MO) was prepared within 30 min of the isolation procedure. All buffers were placed in a 37.5°C water bath on the day of the isolation. Stop buffer was made with 5% FBS in perfusion buffer on the day of isolation. Mice were anesthetized using isoflurane, and the chest was opened to expose the heart for perfusion with 7 mL of EDTA buffer via the right ventricle. The heart was removed by cutting the vasculature above the level of the thymus and immediately placed in a beaker of ice‐cold perfusion buffer. The heart was then transferred to a cooling chamber containing perfusion buffer for the removal of connective tissue surrounding the ascending aorta. The aorta was dissected close to the brachiocephalic artery and ligated to a perfusion buffer‐filled aortic cannula. The cannulated heart was transferred to the Langendorff apparatus and perfused at a rate of 4 mL/min for 4 min with perfusion buffer, followed by a second perfusion with digestion buffer at the same rate for 20 min. The heart was then transferred into a 30‐mm dish containing collagenase buffer to remove excess tissue, including the right ventricular wall and atria. The remaining tissue, including left ventricular wall and septum, was transferred into a new 30‐mm dish containing ~5 mL of collagenase buffer and gently separated into 1‐mm pieces using fine forceps before being gently triturated for ~2 min, using a trimmed 1‐mL pipette tip to ensure a larger bore. Afterwards, 5 mL of stop buffer was added to the dish for a second round of trituration for ~1 min. The generated cell suspension was passed through a 100‐μm filter into a 50‐mL conical tube, and the filtered suspension was transferred to a 15‐mL conical tube for Ca^2+^ reintroduction. Cells underwent four sequential rounds of Ca^2+^ reintroduction (0.25, 0.5, 0.75, and 1 mM) in perfusion buffer by gravity settling for 20 min/round. After the final round of Ca^2+^ reintroduction, the cells were resuspended in 1–3 mL of Tyrode's solution containing (in mM) 140 NaCl, 5 KCl, 2 CaCl_2_, MgCl_2_, 10 HEPES, and 5.6 glucose (adjusted to pH 7.4 with NaOH).

#### 2.1.9 Immunofluorescence

A resuspension of cardiomyocytes in Tyrode's solution was added to a 12‐well plate, with each well containing a 10‐μg/mL laminin‐coated coverslip. The cardiomyocytes were then incubated at 37.5°C for 1 h, followed by fixation and permeabilization with PBS containing 4% paraformaldehyde (PFA) and 0.1% Triton X‐100 at 37.5°C for 15 min. The cells were then blocked with 5% goat serum in PBS containing 0.2% Tween‐20 (PBS‐T) for 20 min at RT, followed by overnight incubation at 4°C in blocking buffer containing primary antibodies against α‐actinin (sarcomeric) (cat. # A7732, 1:100, Sigma‐Aldrich, St. Louis, MO) and titin (cat. # 27867‐1‐AP, 1:200, Proteintech, Rosemont, IL). Following several rinses with PBS, the cells were incubated in PBS containing 1% BSA and Alexa Fluor‐488 and DyLight‐594‐conjugated goat secondary antibodies (cat. # A32723 and 35,561, respectively, 1:1000, Thermo‐Fisher Scientific, Waltham, MA) for 1 h at RT. The cells were then washed several times and allowed to dry before mounting the coverslips on microscope slides, using Vectashield Antifade mounting medium containing DAPI (cat. # H‐1000‐10, Vector Laboratories Inc., Newark, CA). The edges of the coverslip were sealed with clear nail polish. The slides were then imaged at 100x using an Olympus SPIN SR‐10 Spinning Disc Confocal microscope (IXplore, Olympus Corp., Breinigsville, PA). Cells were selected for imaging based on rectangular, rod‐like morphology. Cells with rounded edges or membrane blebbing were excluded. For Z‐dimension image acquisition, 1‐μm steps were used and 10–15 cells/mouse were imaged. Cell length and width, as well as sarcomere length and Z‐disc thickness, were determined using the line tool in NIH ImageJ software. The images were then judged by three blinded assessors for the presence or absence of sarcomere disorganization based on titin immunolabeling and based on the appearance of “fuzzy” rather than sharp sarcomeres, as well as punctate titin labeling located away from the sarcomere. To examine the relationship between sarcomere disorganization and genotype, a pool of 12 cells for each mouse was randomly selected to calculate the percentage of cells of that pool judged as displaying sarcomere disorganization based on titin immunolabeling.

#### 2.1.10 Quantitative real‐time PCR analysis

Expression levels of *Nppa*, *Col3a1*, *Rgs1*, *Rgs2*, *Rgs3*, *Rgs4*, *Rgs5*, and *Serca* were determined by real‐time PCR using total RNA extracted from the heart apex. Total RNA was isolated using the TRIzol extraction method (Thermo Fisher Scientific, Waltham, MA), with tissue homogenizer tubes and an Omni Bead Ruptor 24 homogenizer (Omni International, Kennesaw, GA). RNA was purified using the Purelink RNA Minikit, according to the manufacturer's instructions (cat. # 12–183‐018A, Thermo Fisher Scientific, Waltham, MA). The following primer probes were used in the real‐time PCR assay with TaqMan gene expression master mix (cat. # 43‐044‐37, Thermo Fisher Scientific, Waltham, MA), as directed by the manufacturer: *Nppa*, Mm01255747_g1; *Col3a1*, Mm00802300_m1; *Rgs1*, Mm00450170_m1, *Rgs2*, Mm01292909_g1; *Rgs3*, Mm01267574_m1; *Rgs4*, Mm00501389_m1; *Rgs5*, Mm00654112_m1; and *Gapdh*, Mm99999915_g1. The ΔC_t_ method (where C_t_ is the threshold cycle) was used to calculate *Nppa*, *Col3a1*, *Rgs1*, *Rgs2*, *Rgs3*, *Rgs4*, and *Rgs5* mRNA expression after normalization to *Gapdh* expression.

#### 2.1.11 Western blot

The heart base of male WT and *Rgs2/5* dbKO mice was homogenized in RIPA lysis buffer containing cOmplete™, Mini Protease Inhibitor Cocktail and PhosSTOP™ (cat. # 4906845001, Sigma‐Aldrich, St. Louis, MO). The homogenate was centrifuged (15,294 *g*, 4°C) for 20 min. The supernatant was collected, and protein concentration was determined by Pierce™ BCA Protein Assay Kit (cat. # FERA65453, ThermoFisher Scientific, Waltham, MA). Tissue lysates were added to Laemmli sample buffer (cat. # 1610737XTU, Bio‐Rad Laboratories, Hercules, CA) and boiled for 15 min, followed by sample loading (20 μg of total protein), protein separation by SDS‐PAGE (12% gel) and transfer to polyvinylidene difluoride (PVDF) membrane (cat. # IPVH15150, Sigma‐Aldrich, St. Louis, MO). The PVDF membranes were blocked with 5% nonfat milk or 5% bovine serum albumin (for phosphorylated proteins) in tris‐buffered saline containing 0.1% Tween (TBS‐T) for 1 hour, followed by overnight incubation on a rocker at 4°C with corresponding Cell Signaling Technology (Cell Signaling Technology, Danvers, MA) primary antibodies against p‐AKT (S473) (cat. #4060S, 1:2000), AKT (pan) (cat. #2920S, 1:2000), p‐p44/42 MAPK (T202/Y204) (D13.14.4E) XP (cat. #4370S, 1:2000), p44/42 MAPK (ERK 1/2) (137FS) (cat. #4695S, 1:1000), P‐GSK‐3β (Ser9) (cat. #9336S, 1:1000), GSK‐3β (cat. #9315S, 1:1000), Phospho‐p70 S6 Kinase (Thr389) (cat. #108D2, 1:1000), p70 S6 Kinase (49D7) (cat. #2708S, 1:1000), pan‐calcineurin A (cat. #2614S, 1:1000), α‐Actinin (D6F6) XP (cat. #6487S, 1:1000), CaMKII (pan) (D11A10) (cat. #4436S, 1:1000), and FLAG® M2 (cat. # F1804, 1:2000, Sigma Aldrich, St. Louis, MO). After several washes with TBS‐T, the appropriate horseradish peroxidase‐labeled secondary antibody (cat. #7074S or 7076S, 1:2000, Cell Signaling) was added and incubated for 2 hours at RT, followed by additional washes with TBS‐T and the application of SuperSignal™ West Pico PLUS Chemiluminescent Substrate (cat. # PI34579, ThermoFisher Scientific, Waltham, MA) for the visualization of protein bands using Azure 600 (Azure Biosystems, Dublin, CA). The membranes were stripped with PVDF membrane stripping buffer (cat. # DSP65000, Dot Scientific, Burton, MI), re‐blocked, and incubated with primary antibodies against loading controls GAPDH (cat. #97166, 1:2000, Cell signaling Technology) or β‐Actin (cat. #4967S, 1:1000, Cell signaling Technology). Densitometric analysis of the protein bands was performed with NIH ImageJ software.

#### 2.1.12 Data analysis and statistics

All values are presented as mean ± SD. All statistical analysis was performed using GraphPad Prism 10 software. Two‐way ANOVA mixed model was performed to determine the effect of genotype and treatment and significant interaction within and between groups. If the ANOVA yielded a significant effect of a factor or a significant interaction between multiple factors, Šidák post hoc analysis was used to determine the significance of differences between groups. For statistical analysis of tissue fibrosis scoring, we used nonparametric ANOVA with Kruskal–Wallis test, followed by a two‐stage linear step‐up procedure of Benjamini, Krieger, and Yekutieli for multiple comparisons. A *P* value of <0.05 was considered statistically significant.

## 3 RESULTS

### 3.1 Loss of RGS2 and/or RGS5 results in left ventricular dilation and dysfunction

**FIGURE 1:**
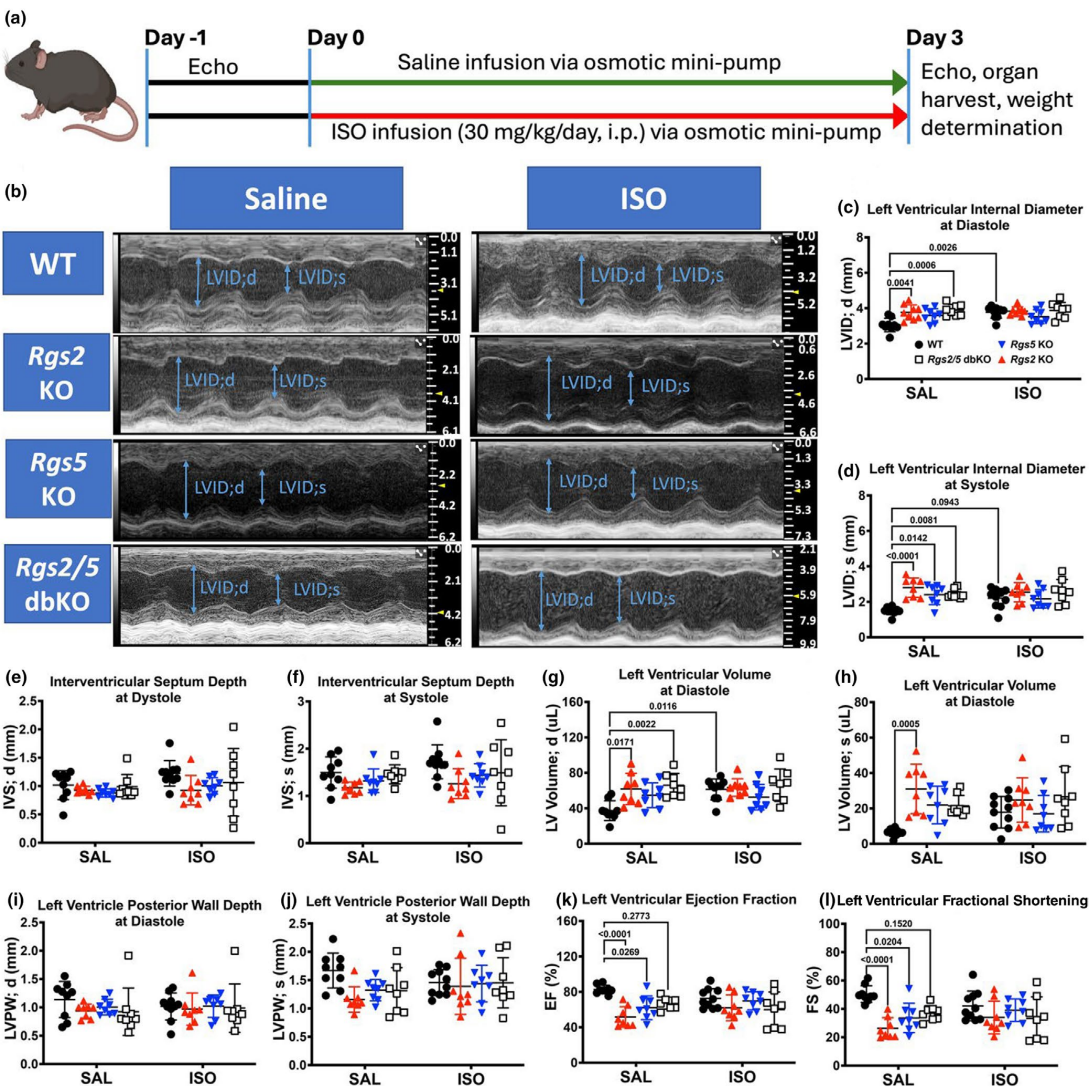
Loss of RGS2 or RGS5 leads to left ventricular dilation and reduced left ventricular (LV) function. (a) Schematic illustration of the timeline for non‐invasive cardiac structure and function assessment by echocardiography (echo), systemic infusion of saline or isoproterenol (ISO) in conscious mice, organ harvest, and measurement of heart weight and tibia length after euthanasia. (b) Representative parasternal long‐axis M‐mode LV echocardiograms at mid‐ventricular level, with representative measurements of left ventricular internal diameter at diastole (LVID; d) and systole (LVID; s). Mice (*n* = 8–14 per group) received subchronic (3 days) infusion of saline or isoproterenol (ISO, 30 mg/kg/day) via osmotic mini‐pumps (schema in a). Echocardiography was performed under anesthesia (isoflurane) 72 h after mini‐pump implantation. Measurement of chamber size at end‐diastole and end‐systole, internal diameter (c and d, respectively), and volume (g and h) reveals LV dilation due to the loss of either RGS2 or RGS5. ISO infusion induced an increase in end‐diastole chamber size in WT mice. No significant differences in septum (e and f) or posterior wall (i and j) thickness due to treatment or genotype were observed. Left ventricular function, as quantified by ejection fraction (%EF, k) and fractional shortening (%FS, l), was reduced due to the loss of either RGS2 or RGS5. Symbols in the graphs represent individual samples from separate animals (*n* = 8–12 mice per group). Data in the summarized plots are mean ± standard deviation. A two‐way ANOVA mixed model, with Šidák post hoc analysis, was applied for multiple comparisons for statistical significance. Stated numbers in the graphs are *p*‐values for individual comparisons. SAL, saline; ISO, isoproterenol.

We previously reported that male *Rgs2/5* dbKO mice, unlike their female counterparts, display left ventricular dilation at baseline and developed arrhythmias related to increased G_i/o_ activity (Dahlen et al., 2022). Here, we postulated that enhanced β_2_AR – G_i/o_ signaling due to acute elevation of circulating norepinephrine exacerbates LV remodeling in mice lacking RGS2 and/or RGS5. To test this hypothesis, we challenged adult male wild‐type (WT), *Rgs2* KO, *Rgs5* KO, and *Rgs2/5* dbKO mice with 3 days of βAR stimulation via systemic ISO infusion (Figure 1a). In saline‐infused mice, LV chamber size was enlarged in *Rgs2* KO, *Rgs5* KO, and *Rgs2/5* dbKO hearts, as determined by internal diameter and volume at systole, while at diastole, LV chamber size was enlarged only in *Rgs2* KO and *Rgs2/5* dbKO compared to WT hearts (Figure 1b,c,d,g and h). In addition, percent ejection fraction (%EF) and fractional shortening (%FS) in both *Rgs2* KO and *Rgs5* KO mice were reduced (Figure 1k,l). As previously reported (Dahlen et al., 2022), *Rgs2/5* dbKO hearts showed LV dilation relative to WT hearts in saline‐infused mice at diastole and systole and increased LV volume at diastole (Figure 1b–d,g). However, unlike in single KO mice, the differences in %EF and %FS between *Rgs2/5* dbKO and WT mice showed a trend but did not reach statistical significance (Figure 1k, l). Interestingly, LV dilation in *Rgs2* KO, *Rgs5* KO, and *Rgs2/5* dbKO mice was not accompanied by any observable changes in septum or posterior wall thickness compared to WT mice (Figure 1b,e,f,i,j). Subchronic (3 days) ISO infusion increased LV chamber size only in WT but not in any of the KO genotypes (Figure 1b,c,g). These data suggested that the absence of either RGS2 or RGS5 alone leads to LV structural remodeling, and that *Rgs5* deletion seems to blunt LV remodeling in *Rgs2/5* dbKO, as the remodeling appeared to be most severe in *Rgs2* KO after saline treatment.

### 3.2 ISO infusion does not affect compensatory increases in *Rgs5* expression upon deletion of *Rgs2* and *Rgs4* expression upon deletion of *Rgs5*

**FIGURE 2:**
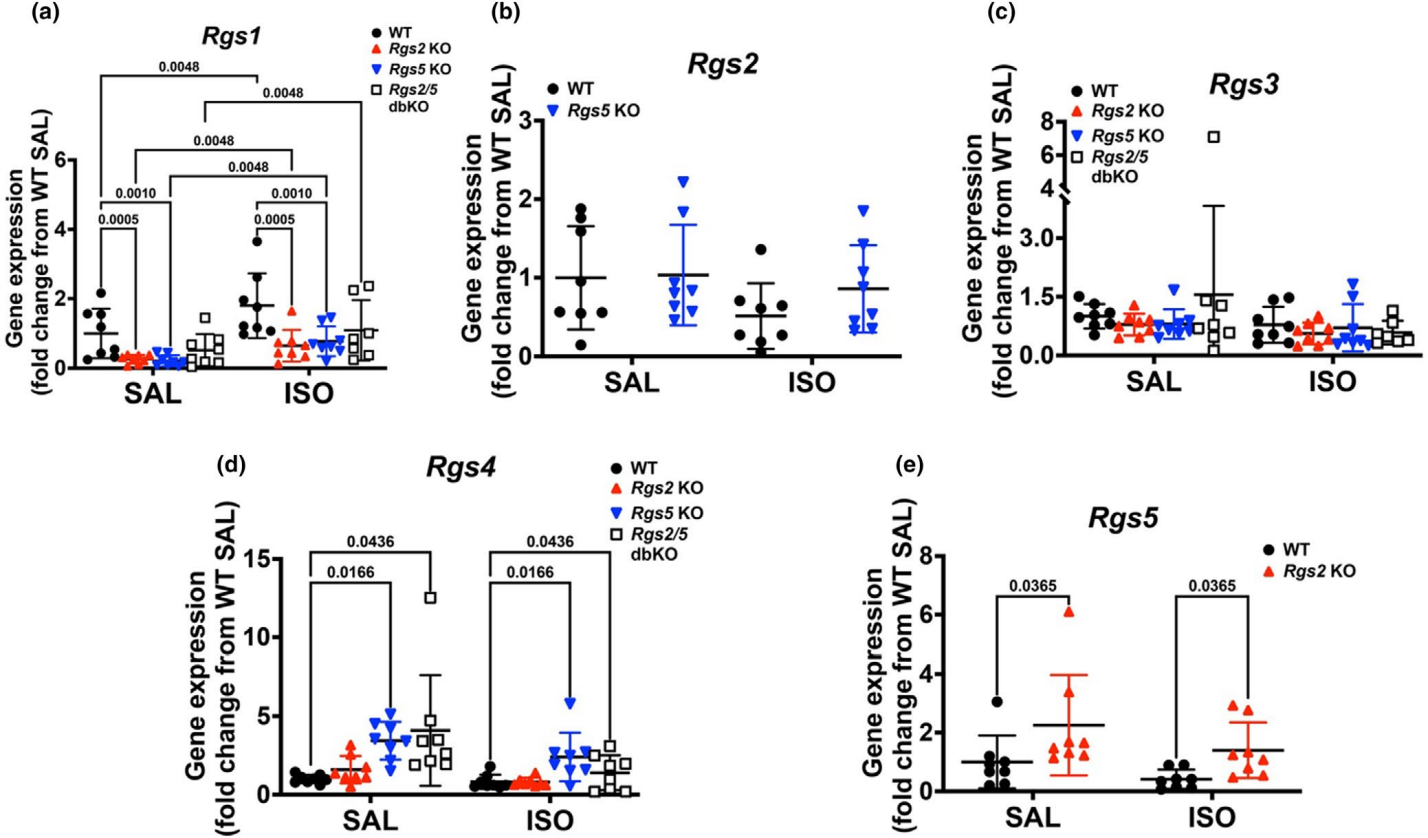
Deletion of *Rgs2 and 5* induces differential expression of other R4 members in the ventricular myocardium. To examine any potential compensatory mechanisms that occur due to the loss of RGS2 and/or RGS5, we performed quantitative PCR analysis to determine mRNA expression of different members of the R4 protein family that have previously been identified as having cardiac expression. (a) Expression of *Rgs1* is significantly decreased compared to WT due to the loss of either RGS2 or RGS5 but not both. Regardless of genotype, ISO infusion increased the expression of *Rgs1*. (b) Expression of *Rgs2* is not influenced by the loss of *Rgs5* or by ISO infusion. (c) Expression of *Rgs3* was not influenced by the loss of RGS2 and/or RGS5, regardless of saline or ISO treatment. (d) Expression of *Rgs4* was significantly increased due to the loss of RGS5, and this difference was unchanged by ISO infusion. (e) Expression of *Rgs5* was significantly increased due to loss of RGS2 and was unaffected by ISO infusion, regardless of genotype. Symbols in the graphs represent individual samples from separate animals (*n* = 8 for each genotype and treatment). mRNA expression is expressed as a fold change from WT SAL or ISO control, after normalizing the expression of each gene by the expression of the housekeeping gene, *Gapdh*. Data in the summarized plots are mean ± standard deviation. A two‐way ANOVA mixed model, with Šidák post hoc analysis, was applied for multiple comparisons for statistical significance. Stated numbers in the graphs are p‐values for individual comparisons. SAL, saline; ISO, isoproterenol.

Because we observed differences in the severity of LV remodeling among the three KO genotypes relative WT, we used quantitative PCR to assess ventricular tissue mRNA to determine how the deletion of *Rgs2*, *Rgs5*, or both alters the expression of other members of the R4 family, including *Rgs1*, *Rgs3*, and *Rgs4*, that are reported to be prominently expressed in the myocardium (Riddle et al., 2005). In saline‐infused WT mice, the order of mRNA expression of the five R4 *Rgs* genes was *Rgs5* > *Rgs2* > *Rgs3 > Rgs4* >>> *Rgs1* (Figure 2a–e). *Rgs1* expression was <30x less, relative to the expression of the other R4 family members. Interestingly, however, *Rgs1* expression was markedly reduced in ventricular tissue from *Rgs2 KO and Rgs5* KO but not from *Rgs2/5* dbKO mice (Figure 2a). ISO infusion proportionally increased *Rgs1* mRNA expression in ventricular tissue from all genotypes, thus maintaining the reduced expression in *Rgs2* KO and *Rgs5* KO tissues, relative to WT tissues (Figure 2a). The expression of *Rgs2* mRNA was unchanged in *Rgs5* KO tissue but trended low in WT tissue, following ISO infusion (Figure 2b). In contrast, *Rgs3* mRNA expression was similar among all genotypes and was unaffected by ISO infusion (Figure 2c). The expression of *Rgs4* mRNA, in contrast to *Rgs1* mRNA expression, was significantly elevated in tissues from saline infused *Rgs5* KO and *Rgs2/5* dbKO mice and was not affected by ISO infusion (Figure 2d). Interestingly, *Rgs5* mRNA expression increased ~5 fold in *Rgs2* KO relative to WT tissue. Although the elevated *Rgs5* mRNA expression levels between WT and *Rgs2* KO tissues persisted after ISO infusion, the difference decreased slightly after drug infusion (Figure 2e). Together, these data indicated that in the ventricular myocardium, deletion of *Rgs5* or dual deletion of *Rgs2* and *5* induces a compensatory increase in *Rgs4* mRNA expression, whereas *Rgs2* deletion induces a compensatory increase in *Rgs5* expression, all occurring independently of sustained βAR stimulation.

### 3.3 Subchronic ISO infusion induces mild eccentric cardiac remodeling in *Rgs2/5* dbKO mice

**FIGURE 3:**
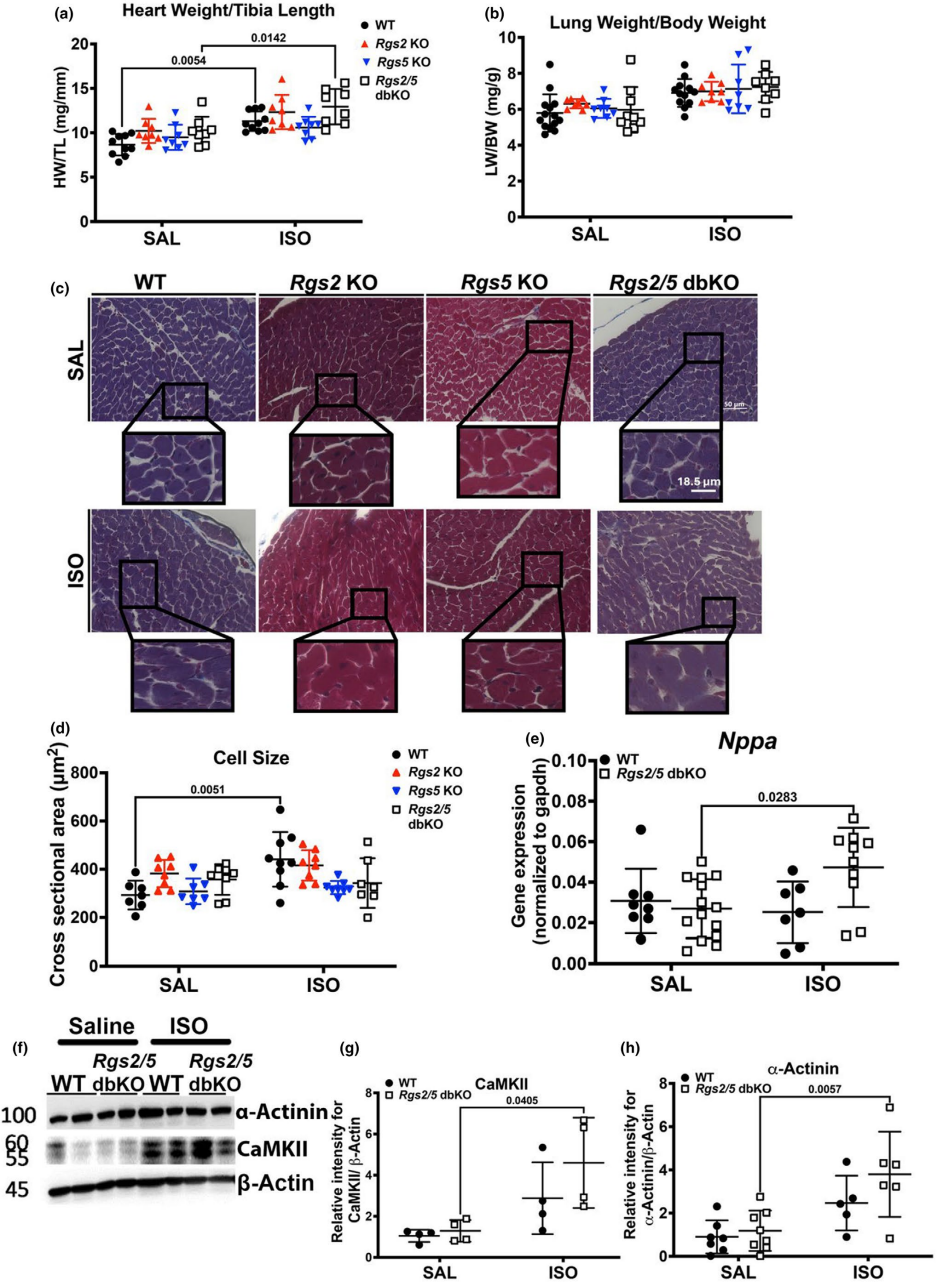
Dual loss of RGS2 and RGS5 increases susceptibility to subchronic ISO infusion‐induced eccentric cardiac hypertrophy. (a, b) Gravimetric analysis of changes due to saline and ISO infusion in heart and lung weight, respectively. Heart samples were harvested shortly after echocardiography at the end of ISO infusion on day 3. (c) Representative images of trichrome‐stained midventricular tissue sections used to determine cross‐sectional cardiomyocyte area. The scale bar in the low magnification image denotes 50 μm, whereas the one in the high magnification denotes 18.5 μm. (d) Summary graph of quantified cross‐sectional area of midventricular cardiomyocytes in saline (SAL)‐ and isoproterenol (ISO)‐treated ventricular tissue sections. (e) Quantitative PCR analysis of changes in *Nppa* mRNA expression in SAL‐ and ISO‐treated ventricular tissue from WT (closed circles) and *Rgs2/5* dbKO (open squares) mice. (f) Representative Western blot for α‐actinin and CaMKII, with summary plots in (g and h), respectively, of SAL‐ and ISO‐treated ventricular tissue from WT (closed circles) and *Rgs2/5* dbKO (open squares) mice. Symbols in the graphs represent individual samples from separate animals (*n* = 8–14 for each genotype and treatment). Data in the summarized plots are mean ± standard deviation. A two‐way ANOVA mixed model, with Šidák post hoc analysis, was applied for multiple comparisons for statistical significance. Stated numbers in the graphs are *p*‐values for individual comparisons.

**FIGURE 4:**
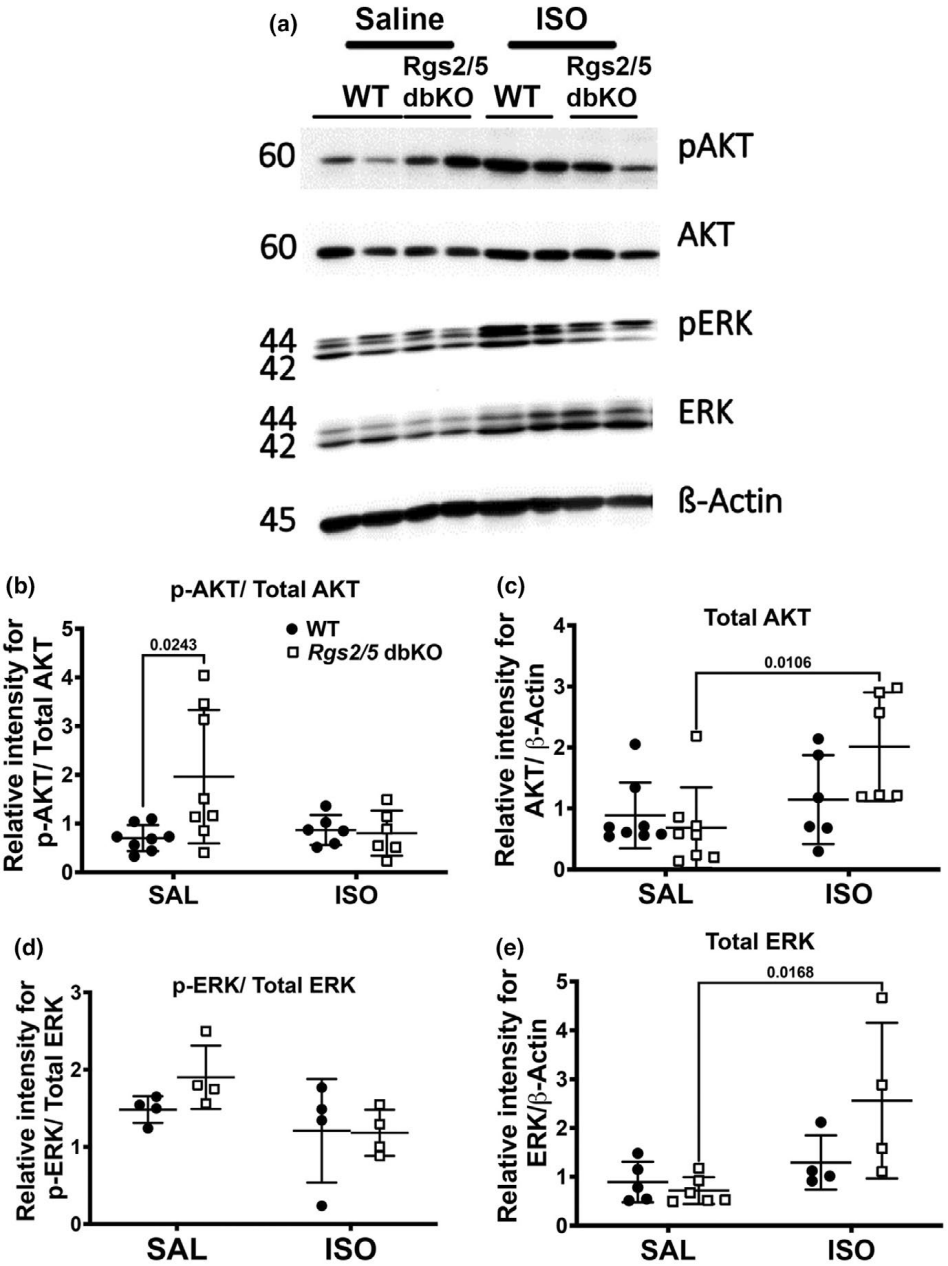
Phosphorylation of AKT but not ERK is increased due to the dual loss of RGS2 and RGS5. (a) Representative Western blot for pAKT, AKT, pERK, and ERK expression in heart base tissue harvested from WT and *Rgs2/5* dbKO mice. (b, d) Quantification of p‐AKT/AKT and p‐ERK/ERK expression, respectively, demonstrating increased phosphorylation of AKT but not ERK in *Rgs2/5* dbKO saline‐treated mice. (c, e) Quantification of total AKT or total ERK expression relative to β‐Actin, respectively. Symbols in the graphs represent individual samples from separate animals (*n* = 8 for WT and *Rgs2/5* dbKO for p‐AKT/AKT, and *n* = 4 for WT and *Rgs2/5* dbKO for p‐ERK/ERK). Data in the summarized plots are mean ± standard deviation. A two‐way ANOVA mixed model, with Šidák post hoc analysis was applied for multiple comparisons for statistical significance. Stated numbers in the graphs are *p*‐values for individual comparisons. SAL, saline; ISO, isoproterenol.

**FIGURE 5:**
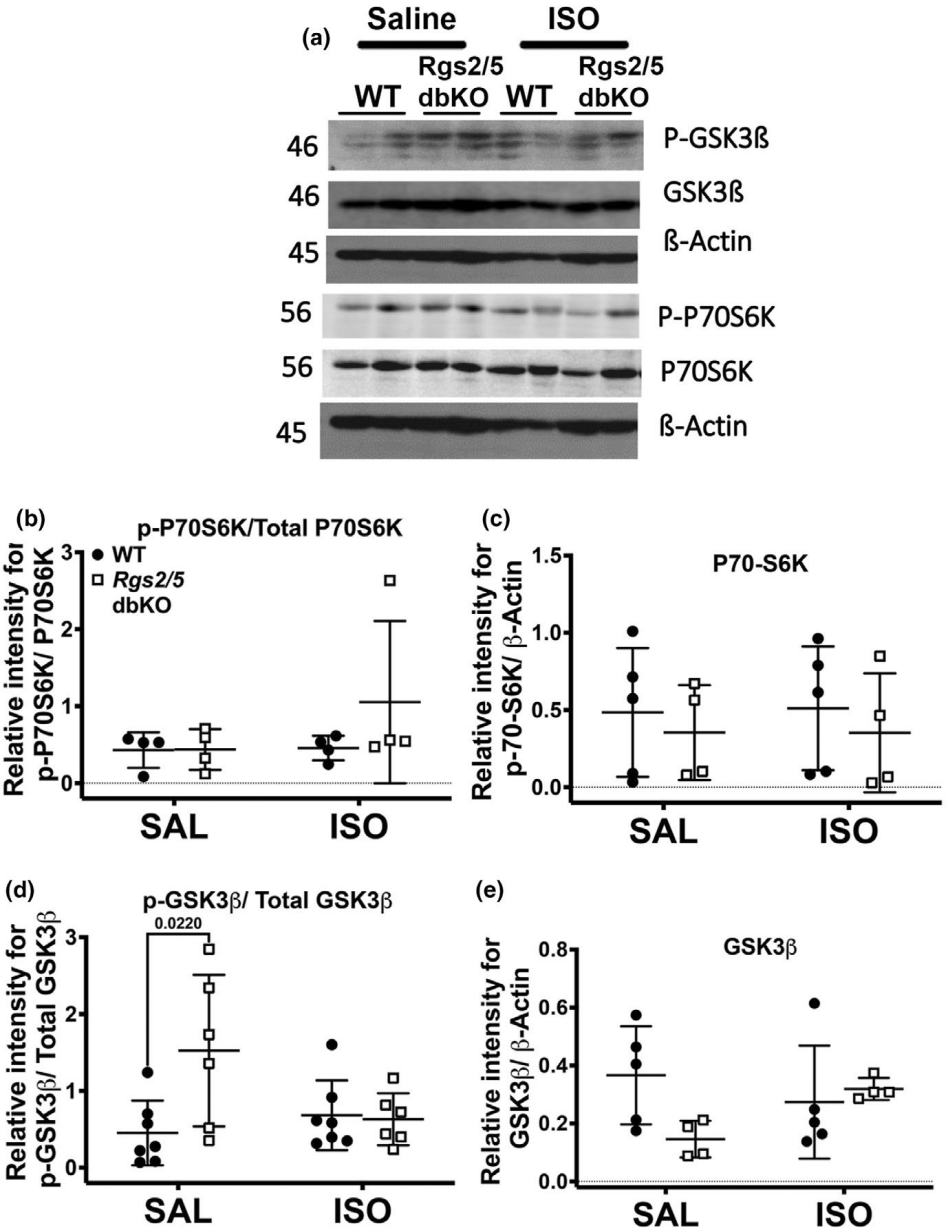
Loss of RGS2 and RGS5 induces increased basal GSK3β phosphorylation. (a) Representative Western blot for p‐GSK3β, GSK3β, p‐P70S6K, and P70S6K expression in heart base tissue harvested from WT and *Rgs2/5* dbKO mice. (b, d) quantification of p‐P70S6K/P70S6K and *p*‐GSK3β/GSK3β expression, respectively, demonstrating increased phosphorylation of GSK3β but not P70S6K in *Rgs2/5* dbKO saline‐treated mice. (c, e) Quantification of total GSK3β or total P70S6K expression relative to β‐Actin, respectively. Symbols in the graphs represent individual samples from separate animals (*n* = 4–5 for each genotype and treatment). Data in the summarized plots are mean ± standard deviation. Two‐way ANOVA mixed model, with Šidák post hoc analysis, was applied for multiple comparisons for statistical significance. Stated numbers in the graphs are p‐values for individual comparisons. SAL, saline; ISO, isoproterenol.

Because ISO infusion increased LV chamber size at diastole in WT mice to similar sizes in saline‐infused knockout mice (Figure 1), we performed gravimetric analysis on both heart and lung tissues to further characterize ISO‐induced cardiac remodeling in comparison to LV remodeling resulting from *Rgs* gene silencing. As shown in Figure 3, there was no difference in normalized heart weight (HW/TL) following saline infusion or due to genotype. However, ISO infusion equally increased HW/TL in both WT and *Rgs2*/5 dbKO mice relative to their respective saline controls (Figure 3a). There was no difference in HW/TL following ISO infusion in either *Rgs2* KO or *Rgs5* KO mice. Additionally, we did not observe any significant difference in lung weight‐to‐body weight ratio due to genotype or treatment (Figure 3b). To further investigate the increase in HW/TL upon ISO infusion without worsening of LV dilation in *Rgs2*/5 dbKO mice, we assessed cross‐sectional area of trichrome‐stained cardiomyocytes from midventricular tissue sections. Cardiomyocytes in ventricular tissue with clearly defined edges and identifiable nuclei were selected for cell dimensional analysis. We observed no significant difference in cell cross‐sectional area due to genotype in saline‐infused mice. However, there was a significant increase in cardiomyocyte cross‐sectional area in cardiomyocytes from ISO‐infused WT mice (Figure 3c,d). Despite the increase in HW/TL upon ISO infusion, cardiomyocyte cross‐sectional area in ventricular tissue from ISO‐infused *Rgs2/5* dbKO mice was unchanged (Figure 3c,d), suggesting that the dual deletion of *Rgs2* and *Rgs5* causes decompensated cardiac remodeling as opposed to ISO‐induced maladaptive concentric hypertrophy in WT mice. To examine this possibility, we performed quantitative PCR using total RNA extracted from ventricular tissue of WT and *Rgs2/5* dbKO mice to assess the expression of *Nppa*, a component of the cardiac fetal gene program considered a biomarker for maladaptive cardiac remodeling (Olson & Schneider, 2003). Interestingly, while *Nppa* mRNA expression was similar in tissues from saline‐treated WT and *Rgs2/5* dbKO mice, ISO infusion induced a robust increase in expression only in tissue from *Rgs2/5* dbKO mice (Figure 3e). ISO infusion also induced a marked increase in the expression of two proteins that are well established to be associated with maladaptive cardiac hypertrophy and heart failure (Anderson et al., 2011; Sheng et al., 2016), including sarcomeric protein, α‐actinin (Figure 3f,g), and the serine–threonine kinase, Ca^2+^‐/calmodulin‐dependent protein kinase II (CaMKII; Figure 3f,h). To further investigate the differences in the effect of ISO on cardiac remodeling between WT and *Rgs2/5* dbKO mice, we examined the cardiac expression and phosphorylation of protein kinase B (AKT) and extracellular signal‐regulated kinase (ERK) which are, respectively, associated with physiologic and pathologic cardiac hypertrophy and both of which are downstream effectors of G_i/o_ signlaing (Anger et al., 2007; Proud, 2004; Yart et al., 2002). In saline‐infused mice, AKT phosphorylation at serine 473 was elevated in *Rgs2/5* dbKO compared to WT (Figure 4a,b). ERK1/2 phosphorylation trended high in saline‐infused *Rgs2/5* dbKO mice but did not reach statistical significance (Figure 4a,d). ISO infusion increased the expression of total AKT and ERK but only in *Rgs2/5* dbKO and not in WT mice (Figure 4a,c,e). However, phosphorylation of glycogen synthase kinase 3β (GSK3β) at serine 9, which is directly downstream of p‐AKT (Abeyrathna & Su, 2015), was augmented in cardiac tissue from saline‐treated *Rgs2/5* dbKO relative to WT mice (Figure 5a,d). We did not observe any difference in the expression of phosphorylated (at threonine 389) or total p70 S6 kinase (P70S6K) due to genotype or treatment (Figure 5a–c). These findings are consistent with the hypothesis that the dual loss of RGS2 and RGS5 disinhibits G_i/o_ signaling in cardiac tissue (Dahlen et al., 2022).

### 3.4 Loss of RGS2 increases sarcomere disorganization in left ventricular cardiomyocytes

**FIGURE 6:**
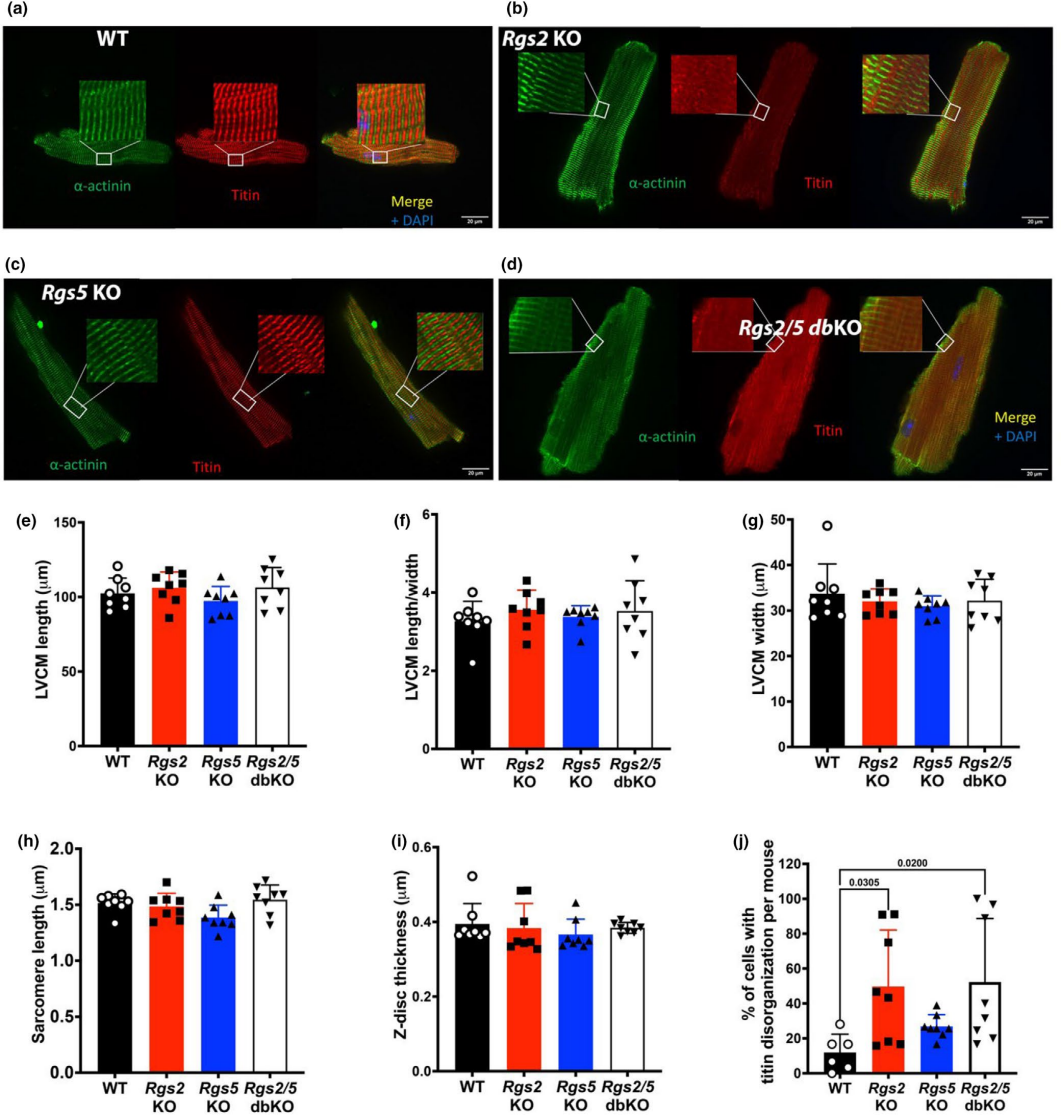
Loss of RGS2 results in increased sarcomere disorganization. (a–d) Representative confocal images of adult LVCM isolated from WT, *Rgs2* KO, *Rgs5* KO, and *Rgs2/5* dbKO mice, where α‐actinin is green and titin is red. Quantitative assessment of (e) cell length, (f) width, (g) width/length ratio, (h) sarcomere length, and (i) Z‐disc thickness are presented by bar graphs for each genotype. (j) Stacked bar graphs showing the ratio of LVCM displaying normal vs. abnormal sarcomere arrangements. The graphs show increased disorganization in LVCM isolated from *Rgs2* KO and *Rgs2/5* dbKO relative to WT mice. Symbols in the graphs represent individual samples from separate animals. Data in the summarized plots are mean ± standard deviation. For e–i, *n* = 8 hearts per group were used, from which at least 100 cells per heart were analyzed, and for J, we quantified sarcomere disorganization based on titin immunolabeling as a percentage of total isolated cells (at least 20 cells per heart) from each mouse. Two‐way ANOVA mixed model, with Šidák post hoc analysis, was applied for multiple comparisons for statistical significance. Stated numbers in the graphs are p‐values for individual comparisons.

To further investigate the reduced LV function due to the loss of RGS2 and RGS5 observed in saline‐treated animals, we performed immunofluorescence labeling of the sarcomeric proteins α‐actinin and titin in freshly isolated LVCM. As shown in Figure 6a–g, the loss of RGS2 and/or RGS5 had no effect on cardiomyocyte length, width, or length/width ratio (Figure 6a–g). Additionally, there was no effect due to genotype on individual sarcomere length or Z‐disc thickness, as measured utilizing α‐actinin labeling (Figure 6a–d,h,i). Unexpectedly, we identified a population of LVCM that displayed an appearance of “disorganized” sarcomere based on titin labeling, as shown in the representative images of cardiomyocytes from *Rgs2* KO and *Rgs2/5* dbKO mice in comparison to the images from WT and *Rgs5* KO mice that display a well‐defined sarcomere organization/arrangement (Figure 6a–d). This structural phenotype was worse in LVCM from *Rgs2* KO and *Rgs2/5* dbKO mice. Approximately 12% and 27% of LVCM from WT and *Rgs5* KO mice, respectively, displayed sarcomere disorganization. In contrast, a significantly higher percentage of LVCM from *Rgs2* KO and *Rgs2/5* dbKO mice showed disorganized sarcomere (~50% and 52%, respectively) in comparison to LVCM WT mice (Figure 6j). These findings suggested that the deficit in LV contractile function due to the loss of RGS2 may be attributable, at least partially, to sarcomere disorganization.

### 3.5 Loss of either RGS2 or RGS5 increases immune cell presence in ventricular tissue

**FIGURE 7:**
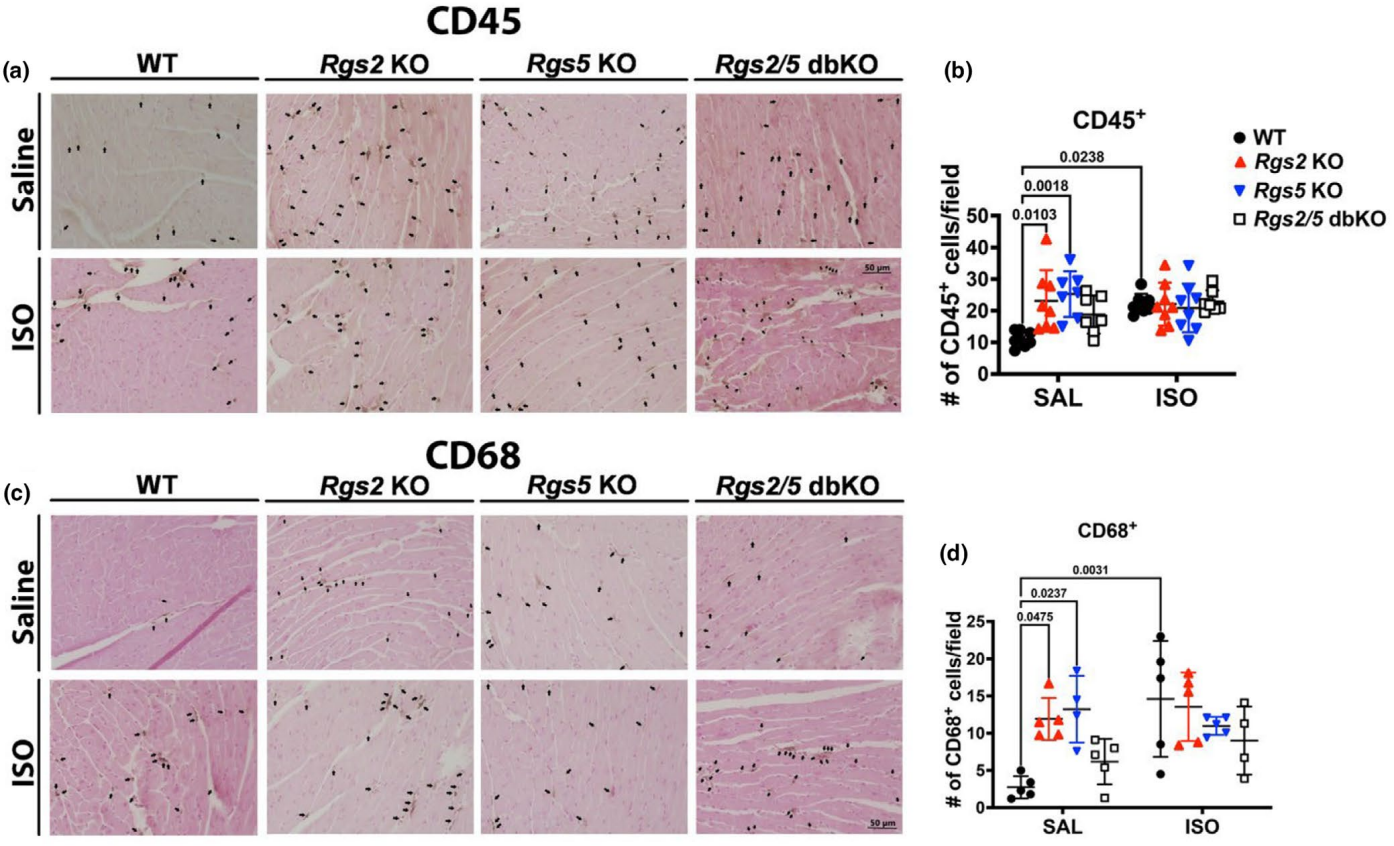
Loss of RGS2 or RGS5 increases immune cell presence in ventricular tissue. Representative CD45 labeling (brown) (a, quantified in b (*n* = 8 hearts)) and CD68 labeling (c, quantified in d (*n* = 4–5 hearts)) demonstrates increased immune cell infiltration in saline‐treated *Rgs2* and *Rgs5* KO ventricular tissue. Symbols in the graphs represent individual samples from separate animals. Data in the summarized plots are mean ± standard deviation. Two‐way ANOVA mixed model, with Šidák post hoc analysis, was applied for multiple comparisons for statistical significance. Stated numbers in the graphs are p‐values for individual comparisons. SAL, saline; ISO, isoproterenol.

Signaling downstream of β_2_AR in noncardiomyocytes in the myocardium, including immune cells, has been shown to play a role in cardiac injury after acute insult (Grisanti et al., 2019; Grisanti, Gumpert, et al., 2016; Grisanti, Traynham, et al., 2016; Tanner et al., 2021). Therefore, we next examined whether the loss of β_2_AR – G_i/o_ signaling regulation due to the dual absence of RGS2 and RGS5 affects immune cell homeostasis in ventricular tissue to contribute to the ensuing structural remodeling. To quantify immune cell presence in midventricular tissue sections, we performed immunohistochemistry to label either total immune cells via CD45 expression or monocyte lineage immune cells via CD68 expression. As expected (Grisanti et al., 2019; Grisanti, Traynham, et al., 2016; Tanner et al., 2021), ISO infusion increased both total immune cell and monocyte lineage immune cell infiltration in WT mice (Figure 7a–d). In knockout tissue sections, there was a markedly elevated presence of both total and monocyte lineage immune cells in midventricular tissue from both *Rgs2* KO and *Rgs5* KO, saline‐treated mice compared to tissue from WT mice. However, ISO infusion did not further increase immune cell infiltration in tissue sections from any of the *Rgs* KO genotypes (Figure 7a–d).

### 3.6 LV contractile dysfunction resulting from the loss of RGS2 and/or RGS5 is not associated with overt cardiac fibrosis or cardiomyocyte apoptosis

**FIGURE 8:**
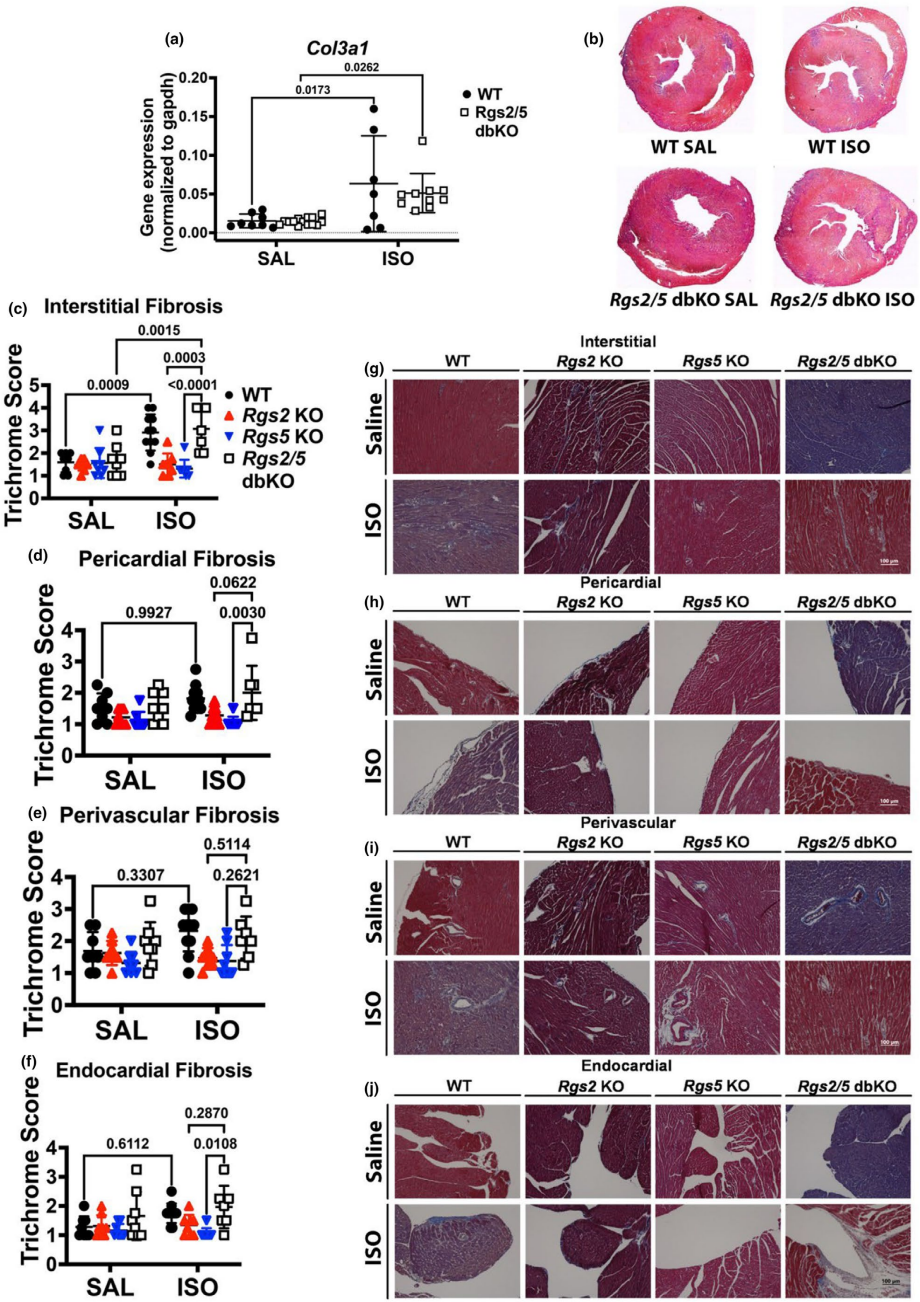
Loss of RGS2 or RGS5 protects against ISO‐induced interstitial and pericardial fibrosis. (b) Representative low (2x)‐magnification photomicrographs of Masson's trichrome stained midventricular cross‐sections from WT and *Rgs2/5* db KO mice infused with saline or ISO for 3 days. (g–j) Representative Masson's trichrome staining, where collagen fibers are stained blue, the cytoplasm is stained red, and nuclei are stained black of cardiac tissue harvested from the vehicle or ISO‐infused mice. Interstitial (quantified in c and represented in g), pericardial (d and h), perivascular (e and i), and endocardial (f and j) fibrosis was scored on an arbitrary scale of 1–4, with 1 displaying little to no fibrosis and 4 displaying extensive fibrosis. ISO infusion significantly increased the amount of interstitial fibrosis present in WT and *Rgs2/5* dbKO tissue but not tissue from *Rgs2* or *Rgs5* KO mice. (a) *Col3a1* expression increased in both WT and *Rgs2/5* dbKO tissue due to ISO infusion. Symbols in the graphs represent individual samples from separate animals. Data in the summarized plots are mean ± standard error. We used nonparametric ANOVA with Kruskal–Wallis test, followed by a two‐stage linear step‐up procedure of Benjamini, Krieger, and Yekutieli for multiple comparisons for the analysis of the fibrosis score. For *Col3a1* gene expression analysis, two‐way ANOVA mixed model, with Šidák post hoc analysis was applied for multiple comparisons for statistical significance. Stated numbers in the graphs are *p*‐values for individual comparisons. SAL, saline; ISO, isoproterenol.

**FIGURE 9:**
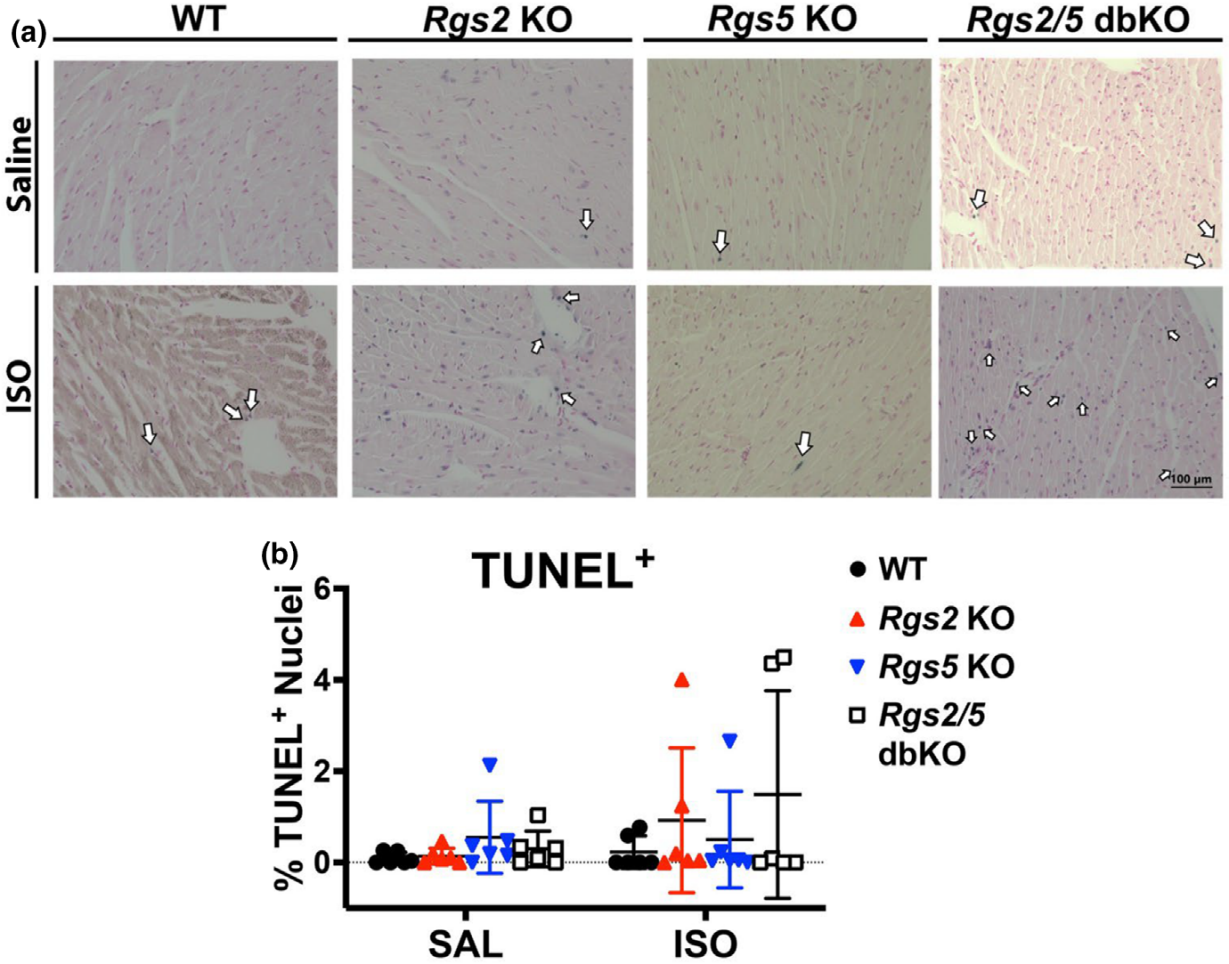
Loss of RGS2 and/or RGS5 does not increase apoptosis in cardiac tissue in either saline or ISO‐infused animals. (a) Representative TUNEL staining, where TUNEL‐positive (TUNEL^+^) nuclei are stained blue. The tissue sections were counterstained with nuclear fast red, which stains TUNEL‐negative nuclei pink. (b) The total number of TUNEL‐positive and ‐negative nuclei was quantified from 10 fields per section and the total percent of TUNEL‐positive nuclei was calculated. Symbols in the graphs represent individual samples from separate animals. Data in the summarized plots are mean ± standard deviation.

Ventricular stiffening due to diffuse collagen deposition by activated fibroblasts can contribute to decreased ventricular function (He et al., 2022). Therefore, we determined whether decreased LV contractile function in *Rgs* KO mice was associated with increased myocardial collagen deposition, by performing Masson's trichrome staining of midventricular tissue sections from saline and ISO‐infused WT, *Rgs2* KO, *Rgs5* KO, and *Rgs2/5* dbKO mice. Interstitial (Figure 8c,g), pericardial (Figure 8d,h), perivascular (Figure 8e,i), and endocardial (Figure 8f, j) fibrosis was scored on an arbitrary scale of 1–4, with 1 displaying little‐to‐no fibrosis and 4 displaying extensive fibrosis. We did not observe any significant differences in fibrosis due to genotype among tissues from saline‐infused mice, suggesting that the reduction in basal ventricular function in *Rgs2* KO and/or *Rgs5* KO mice is not due to fibrogenesis. Interestingly, we observed a substantial increase in interstitial fibrosis following ISO infusion in ventricular tissue from both WT and *Rgs2/5* dbKO mice (Figure 8c,g), accompanied by a similarly enhanced expression of *Col3a1* (Figure 8a). However, we did not observe increased fibrosis in ventricular tissue from either *Rgs2* KO or *Rgs5* KO mice. Finally, we quantified the percentage of apoptotic cells in midventricular tissue from saline and ISO‐infused WT, *Rgs2* KO, *Rgs5* KO, and *Rgs2/5* dbKO mice to test whether apoptotic cell death contributes to the structural and functional LV impairment in single KO mice. Apoptotic cell labeling by TUNEL assay showed no difference in the percentage of TUNEL^+^ cells, based on genotype or treatment (Figure 9a,b). These findings indicated that cardiomyocyte loss from apoptosis does not contribute to LV structural and functional impairment in the absence of RGS2 and/or RGS5.

## 4 DISCUSSION

Regulation of signaling via heterotrimeric G proteins by RGS proteins is an important mechanism for maintaining cardiovascular homeostasis. Gene deletion or the absence of RGS proteins that regulate G_q/11_ and G_i/o_, including RGS2 and RGS5, causes blood pressure disorders, and mutations and gene variants that affect the expression and/or function of RGS2 and RGS5 have been reported to be associated with several disorders of the cardiovascular system (Heximer et al., 2003; Holobotovskyy et al., 2013; Riddle et al., 2006). Since their discovery a few decades ago, multiple RGS proteins have been shown to be prominently expressed in the human myocardium (Riddle et al., 2005). However, whether or how the function of these RGS proteins is coordinated to finetune G protein activity and downstream signaling in the heart to maintain homeostasis is poorly understood. Using mice singly or dually lacking RGS2 and RGS5, we found that despite a compensatory increase in *Rgs5* mRNA expression in the myocardium of *Rgs2* KO mice, both *Rgs2* KO and *Rgs2/5* dbKO male mice exhibited LV dilation at both systole and diastole, and the structural impairment was insensitive to sustained βAR stimulation with systemic infusion of the nonselective agonist, isoproterenol. In addition, sustained βAR stimulation induced eccentric cardiac remodeling in WT mice and increased collagen expression and interstitial fibrosis in WT and *Rgs2/5* dbKO but not in either *Rgs2* KO or *Rgs5* KO mice. However, sustained βAR stimulation failed to enhance basal activity of mitogen‐activated protein kinase (MAPK) or phosphoinositol‐3 kinase (PI3K) signaling downstream of G_i/o_ class G proteins in the myocardium, despite inducing the expression of total CaMKII, AKT, and ERK proteins. We reported previously that suppressed forskolin‐stimulated cAMP generation in ventricular cardiomyocytes from *Rgs2/5* dbKO mice could be restored by inhibiting G_i/o_ with pertussis toxin, indicating augmented activity of G_i/o_ proteins (Dahlen et al., 2022). Thus, the findings in this study lend further support to the prevailing hypothesis that regulation of basal G_i/o_ activity by RGS2 and RGS5 is crucial to maintaining cardiac structure and function.

The observation that all three *Rgs* KO male mice developed LV dilation at baseline is novel and contrasts previous reports of the absence of such abnormal structural remodeling resulting from the deletion of *Rgs2* or *Rgs5* in mice (Cho et al., 2008; Li et al., 2010; Takimoto et al., 2009). The reasons for the discrepancies between earlier reports and current findings are unclear, though it is noteworthy that the age range of the mice used in this study was wide relative to previous studies (Li et al., 2010; Takimoto et al., 2009). We also note differences in the genetic backgrounds of the mice, protocols for echocardiographic assessment of LV chamber dimensions, performing echocardiography in conscious animals vs. under anesthesia, and the type of anesthetic used (Li et al., 2010; Takimoto et al., 2009). Moreover, we reported previously that LV dilation in *Rgs2/5* dbKO mice was observable only in adult mice (Dahlen et al., 2022), suggesting that the manifestation of the morphologic changes in the heart occurs well after embryonic stage and post‐natal cardiac development in mice. Nonetheless, the morphologic and functional changes described earlier and herein for the three *Rgs* knockout genotypes are consistent with the postulation that abnormal G protein signaling is causally involved in nonischemic cardiomyopathy of the left ventricle or dilated cardiomyopathy (DCM) (Gava et al., 2023; Wang et al., 2023). According to this putative mechanism, abnormal LV remodeling due to the loss of G protein regulation as a result of the absence or downregulation of RGS proteins could be a predisposition to arrhythmias, contractile dysfunction, and increased chamber size in the absence of a pathological stress, such as excessive catecholamine release due to sustained sympathoexcitation (Mestroni et al., 1999; Wang et al., 2023; Weintraub et al., 2017). The molecular mechanism that mediates structural features of DCM resulting from the absence of RGS2, RGS5 or both cannot be ascertained from the current study. However, previous studies have shown that G_i/o_ and G_q/11_ are necessary for the canonical Wnt/β‐catenin signaling pathway in Wnt‐stimulated target gene expression (De Vries et al., 1995), and that the proper regulation of Wnt/B‐catenin‐G_i/o_ signaling by RGS proteins is critical to cardiac development and cardiomyocyte differentiation (Ji et al., 2010). Both RGS2 and RGS5 and other members of the R4 family regulate G_i/o_ and G_q/11_ class G proteins, and the results in this study suggest increased basal signaling downstream of G_i/o_, including PI3K and MAPK pathways. Thus, increased activity of G_i/o_ and G_q/11_ could affect the expression of proteins that are key to appropriate cardiac development and cardiomyocyte differentiation. This suggestion is buttressed by the observation that sustained βAR stimulation with ISO led to LV dilation in WT mice but had no effect on the already dilated chamber in the knockout mice. Alternatively, a compensatory increase in *Rgs4* expression, which occurred in *Rgs5* KO and *Rgs2/5* dbKO ventricular tissues, could suppress G_i/o_ signaling, thereby affecting cardiac development in a similar fashion as has been reported previously regarding the effects of RGS19 overexpression on Wnt/B‐catenin‐G_i/o_ signaling (Ji et al., 2010). Further experiments are warranted to test these hypotheses and for a detailed understanding of the precise role of G protein regulation by R4 RGS proteins in DCM etiology. We also observed the largest fraction of cardiomyocytes from *Rgs2* KO mice with sarcomere disorganization, based on titin immunolabeling, suggesting that abnormal sarcomere organization may contribute to the DCM phenotype in the absence of RGS2. Potentially, this could be due to the role that RGS2 specifically plays in cellular stress response, wherein RGS2 inhibits the eukaryotic transcription initiation factor 2 (eIF2), leading to the expression of stress‐associated proteins (Wang & Chidiac, 2019). The absence of RGS2 could possibly lead to failure of the endoplasmic reticulum protein quality control response (Chen et al., 2023). Because sarcomeric proteins such as titin have a relatively high turn‐over rate (Kötter & Krüger, 2022; Rudolph et al., 2019), misfolding of these proteins may result in poor integration of new proteins into the sarcomere and possibly lead to a disorganized sarcomere phenotype as observed in this study.

We found *Rgs1* mRNA, among the R4 transcripts that were quantified in this study, to be the only R4 gene whose expression was significantly elevated by ISO infusion in all genotypes. Interestingly, the expression of *Rgs1* was suppressed only in single knockout mice but not in mice dually lacking RGS2 and RGS5. *Rgs1* is prominently expressed both in innate and adaptive immune cells (Kehrl, 2016), and because of the extremely low *Rgs1* expression in all genotypes (more than 100‐fold less than *Rgs4*, the next minimally expressed gene of the R4 family), we postulated that changes in the expression of this gene following ISO infusion is likely due to increased population of noncardiomyocytes in the myocardium, such as resident immune cells capable of responding to βAR stimulation by increasing tissue infiltration of more immune cells. Consistent with this hypothesis, we observed a divergence in the number of both total immune cells and monocyte lineage immune cells present between ventricular tissues from saline‐treated single knockouts and *Rgs2/5* dbKO mice. Ventricular tissue from saline‐treated *Rgs2* KO and *Rgs5* KO mice contained more immune cells than *Rgs2/5* dbKO tissue, whereas ISO infusion increased immune cell infiltration only in WT tissue to a level similar to those in saline‐treated single knockouts. The requirement for ISO infusion to increase immune cell presence in WT tissue to similar levels in saline‐treated single KO tissue may be a reflection of augmented basal β_2_AR‐linked G_i/o_ activation and Gβγ signaling due to the absence of RGS2 or RGS5, as evidenced in this study by augmented signaling downstream of PI3K. This suggestion is premised on a previous study showing that chemoattractant receptors predominantly couple to G_i/o_ class G proteins, and that signaling via Gβγ subunits plays a crucial role in gradient sensing and directional immune cell migration (Kehrl, 2016). Other studies have reported that neutrophils expressing RGS‐insensitive Gα_i_ subunit display disrupted trafficking, aging, and clearance and that mice harboring these defective neutrophils fail to mount appropriate response to inflammatory insults (Yan et al., 2021). These findings are congruent with previous observations of impaired immune cell trafficking in mice lacking RGS2 (George et al., 2017; Heximer et al., 1997; Lee et al., 2012; Oliveira‐dos‐Santos et al., 2000; Siderovski et al., 1994) or RGS5 (Chan et al., 2018; Cheng et al., 2015; Takata et al., 2008). ISO‐induced immune cell trafficking has been linked directly to β_2_AR activation, as the recruitment of proinflammatory myeloid populations and lymphocytes in response to ISO infusion was shown to be suppressed in chimeric mice receiving bone marrow transplants from β_2_AR KO donors (Tanner et al., 2021). In these chimeric mice, ISO infusion also resulted in decreased cardiomyocyte death, hypertrophy, and interstitial fibrosis, which were somewhat mirrored, at least partially, by our observations in *Rgs2/5* dbKO mice in the current study. Another possibility is that resident immune cells in *Rgs2* KO and *Rgs5* KO ventricular tissues have anti‐inflammatory properties, potentially related to the suppression of *Rgs1* expression. Further studies involving detailed characterization of various immune cell populations will be needed to fully establish the effects of the interactions among various RGS proteins on the immune response to adrenergic stimulation in the ventricular myocardium.

There are several caveats that limit the inferences and interpretation of the findings in this study. Both RGS2 and RGS5 are broadly expressed both in cardiovascular and noncardiovascular cells, and germline deletion of these genes likely triggers some yet unknown compensatory mechanisms that might contribute to or blunt ISO‐induced cardiac structural and functional phenotypes that were studied herein. In addition, the lack of effect of ISO infusion on the structural changes in the heart of the knockout mice could be due to the short time course or the effective dose of ISO that may differ between WT and *Rgs* knockout mice. Additionally, despite increased HW/TL ratio in *Rgs2/5* dbKO mice after ISO infusion, there was no observed difference in cardiomyocyte cross‐sectional area. This discrepancy could be attributed to cardiomyocyte death and replacement by fibrotic tissue that was inadequately detected by TUNEL assay for apoptosis, which was not significantly elevated in *Rgs2/5* dbKO mice. Moreover, the semi‐quantitative method of cardiac fibrosis assessment has the limitation of potential underestimation or overestimation of fibrosis based on the level of tissue sectioning, as the myocardial fibrosis was diffuse and nonuniform. Furthermore, probing of the same PVDF membranes multiple times for Western blot analysis limits the interpretation of how the absence of RGS2 or RGS5 affects the role that target proteins downstream of G_i/o_ signaling play in cardiac dysfunction resulting from germline deletion of *Rgs2* and/or *Rgs5*. Thus, further studies are warranted to investigate other mechanisms of myocardial injury and cell death such as deposition of key collagens such as Col1a1, pyroptosis, and necroptosis that are activatable by ISO infusion (Borodzicz‐Jazdzyk et al., 2023; Eom et al., 2016). Finally, because of the observed sex‐related phenotypic differences in cardiac structure and function resulting from the dual deletion of *Rgs2* and *Rgs5* in our previous study (Dahlen et al., 2022), we only focused on male mice in this study. This is a major limitation of our study and precludes gaining any insight into the potential impact of sex on the pathogenic mechanisms underlying cardiac abnormalities resulting from the deletion of *Rgs2, Rgs5*, or both.

In summary, the findings in this study show that the absence of RGS2 and/or RGS5 causes abnormal LV remodeling associated with dysregulated signaling via G_i/o_ class G proteins, accompanied by enhanced immune cell presence in the myocardium. However, these effects are not sufficient to exacerbate cardiac dysfunction in response to acute elevation of circulating norepinephrine. Future studies utilizing cell type specific *Rgs* KO animals and complementary techniques such as immune cell sorting will enhance the understanding of the multiple mechanisms by which the dynamic expression of RGS proteins of the R4 family is regulated across various tissue types to promote cardiovascular homeostasis.

## FUNDING INFORMATION

This work was supported by NIH R56 DK132859‐01A1, R01 HL139754, and R01 HL174004‐01A1 and Craig H. Neilsen Foundation Grant 382566 to Patrick Osei‐Owusu.

## CONFLICT OF INTEREST STATEMENT

The authors declare no conflict of interest.

## ETHICS STATEMENT

The protocol for all animal experiments in this study was approved by the Institutional Animal Care and Use Committee at Case Western Reserve University (Protocol Number: 2020‐0011).

## Supporting information


Data S1.

## Data Availability

Data that support the findings of this study are available to the editors of the journal for review or query and to the public upon reasonable requests.
