# Supplementary material for: Germline deletion of Rgs2 and/or Rgs5 in male mice does not exacerbate left ventricular remodeling induced by subchronic isoproterenol infusion
Source: Physiol Rep. 2025 Jan 2;13(1):e70178. doi: 10.14814/phy2.70178 (PMC11695115; doi:10.14814/phy2.70178)
Supplement: Supplementary file 1 — Data S1. [file PHY2-13-e70178-s001.docx]

**Supplementary Material**

**Germline Deletion of *Rgs2* and/or *Rgs5* in Male Mice Does Not Exacerbate Left Ventricular Remodeling Induced by Subchronic Isoproterenol Infusion**

Shelby Dahlen^1^, Ipsita Mohanty^2^, Bo Sun^1^, Sanjana Nallapaneni^1^, Patrick Osei-Owusu^1^*

^1^Department of Physiology & Biophysics, Case Western Reserve University School of Medicine, Cleveland, OH, United States of America

^2^Department of Pharmacology & Physiology, Drexel University College of Medicine, Philadelphia, PA, United States of America

Short title: Left Ventricular Dilation Resulting from the Loss of RGS2 or RGS5

**Supplementary Complete Membranes of immunoblots**

**
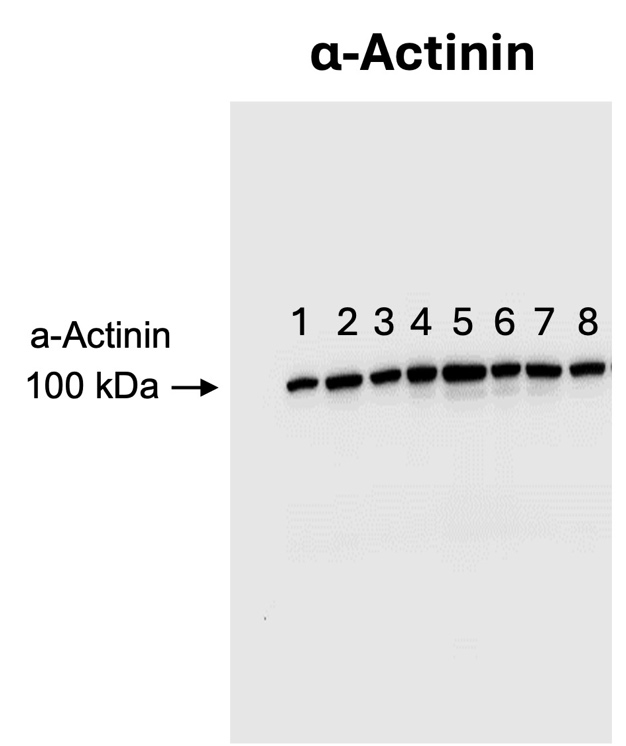
**

**
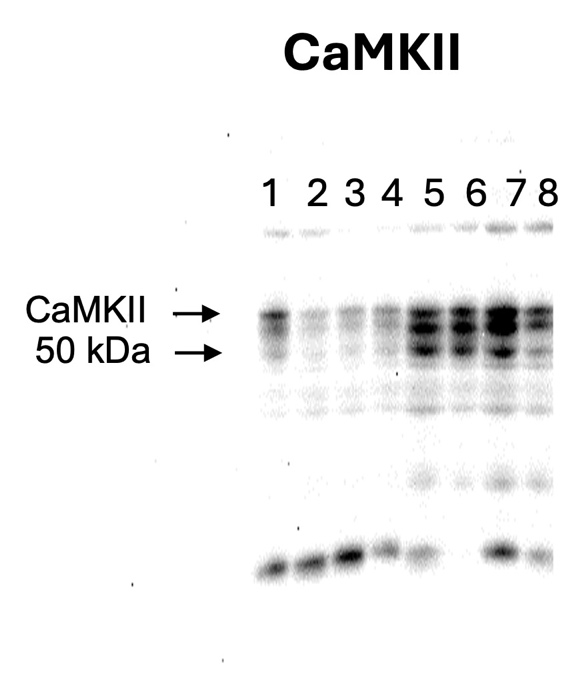
**

**
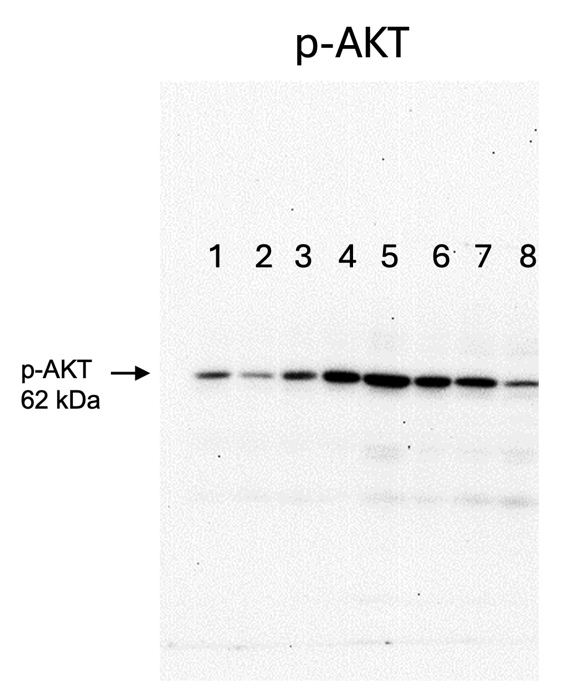
**

**
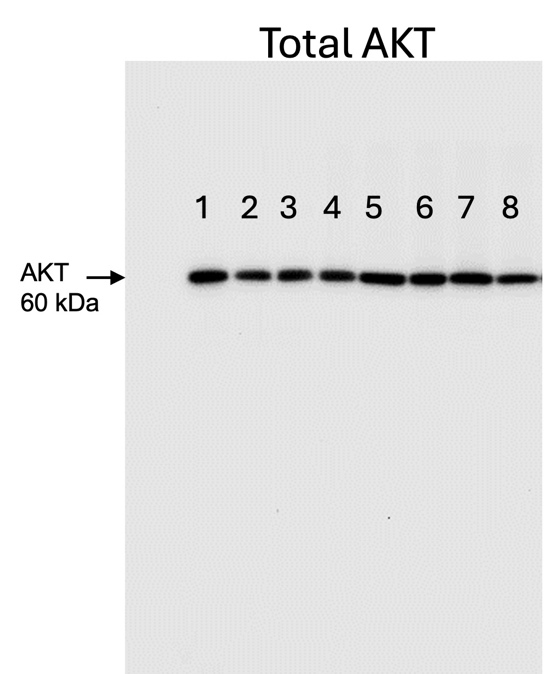
**

**
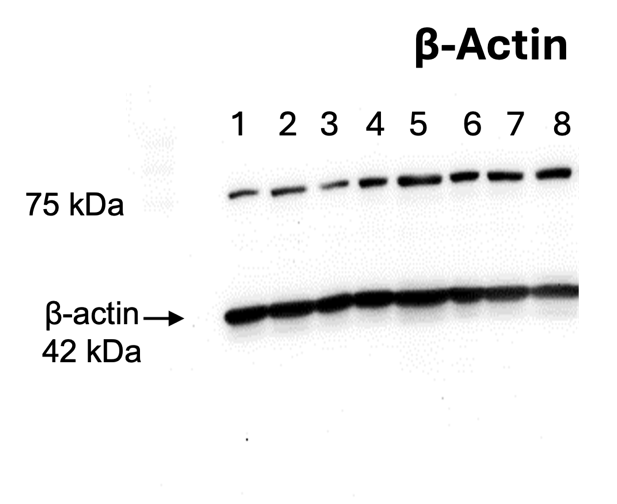
**

**
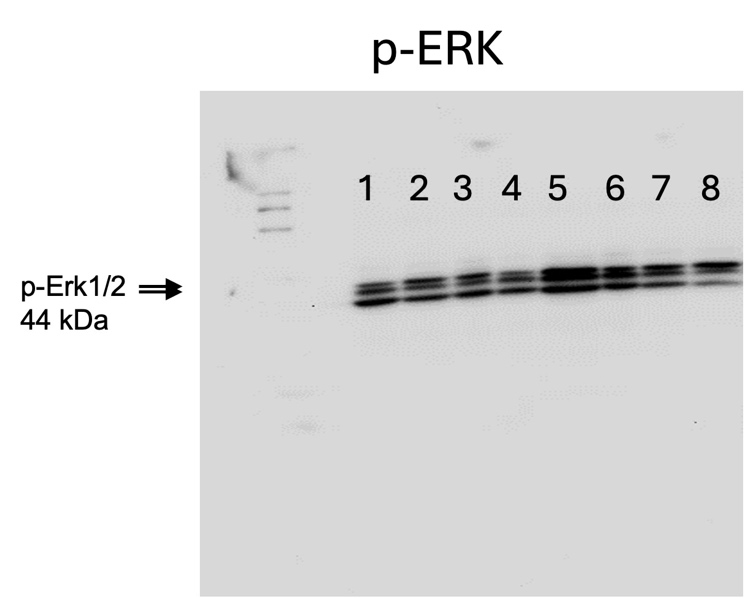
**

**
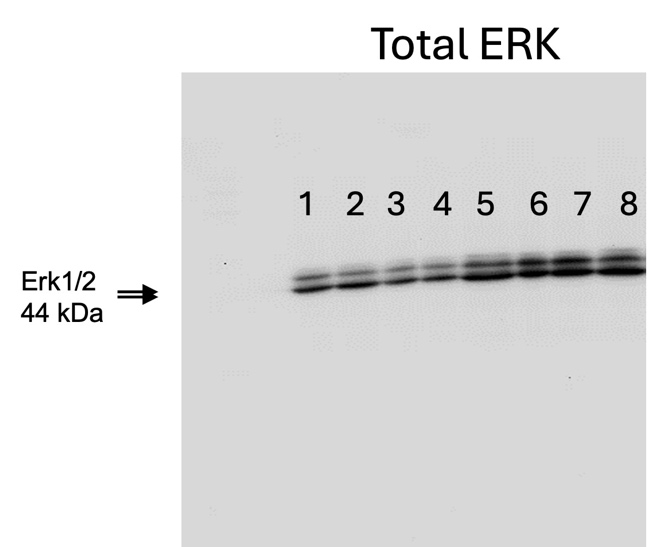
**

**
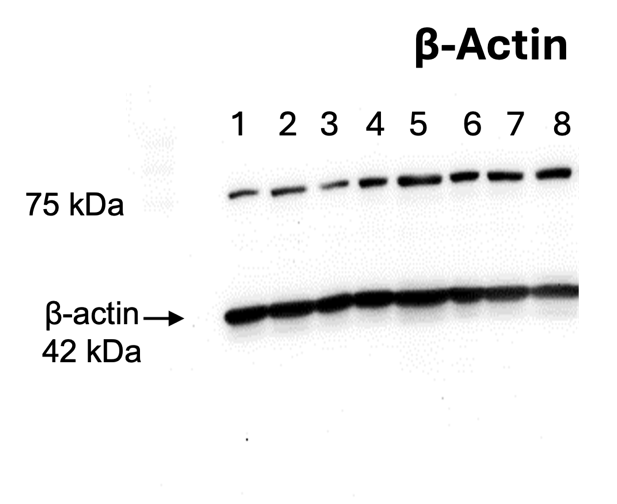
**

**
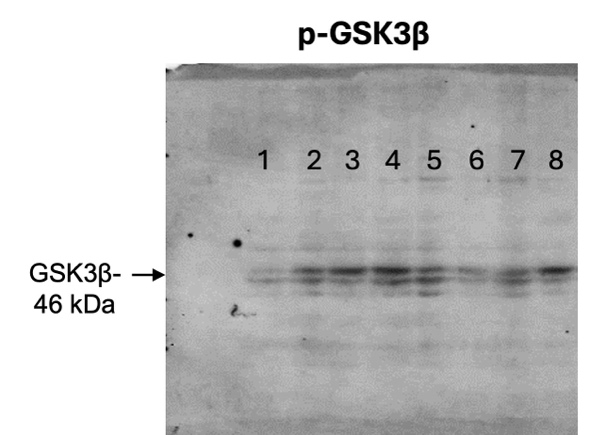
**

**
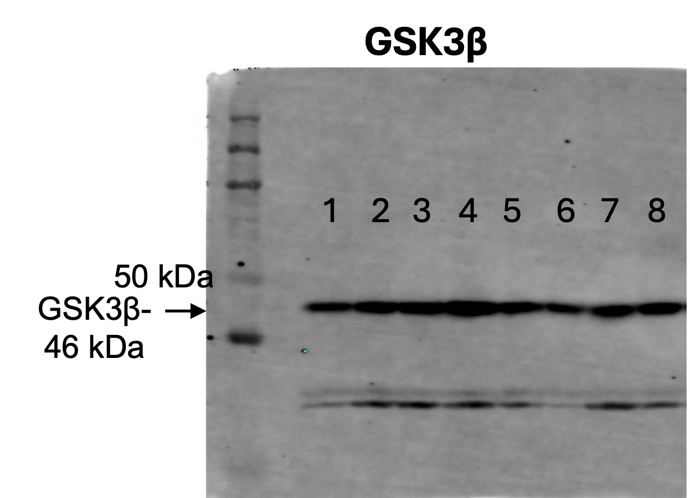
**

**
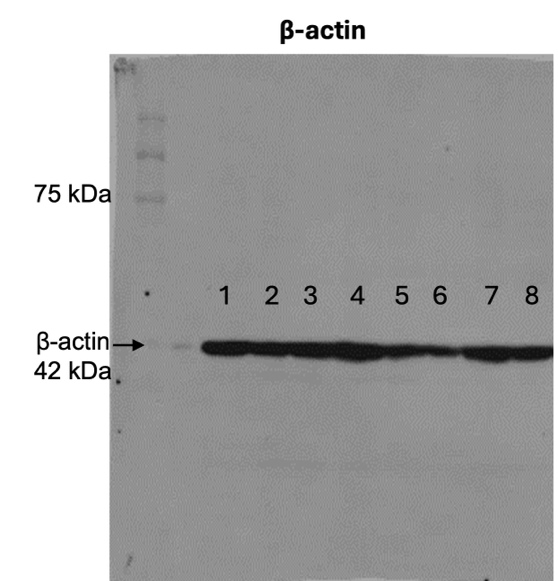
**

**
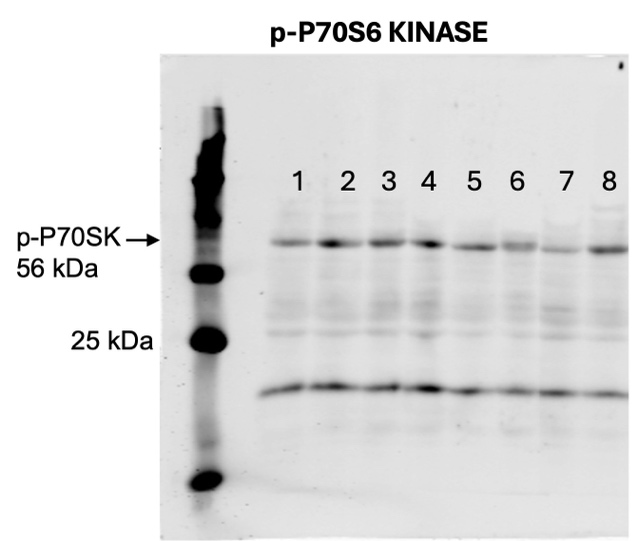
**

**
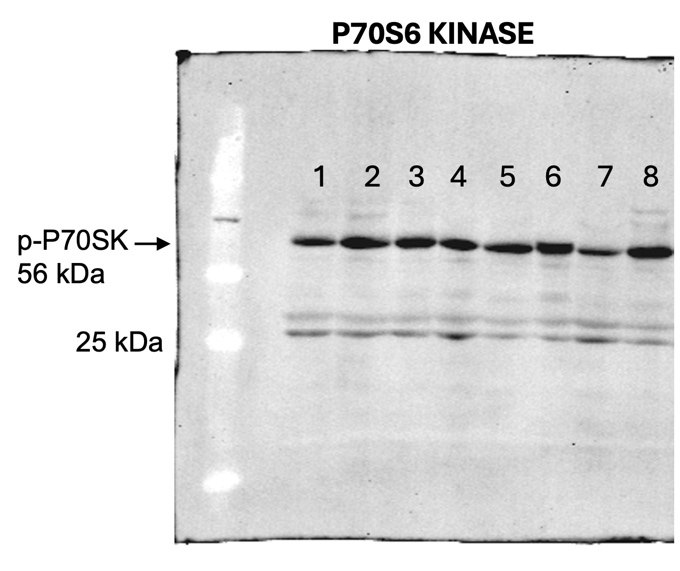
**

**
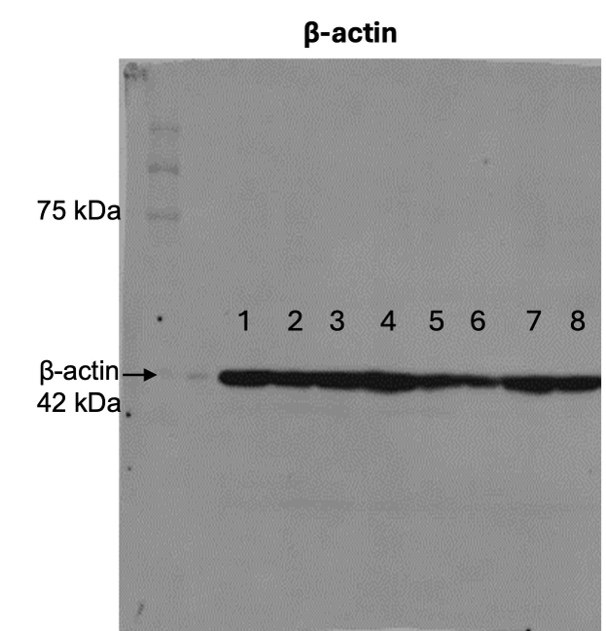
**
